# Mitochondrial superoxide dismutase Sod2 suppresses nuclear genome instability during oxidative stress

**DOI:** 10.1093/genetics/iyad147

**Published:** 2023-08-29

**Authors:** Sonia Vidushi Gupta, Lillian Campos, Kristina Hildegard Schmidt

**Affiliations:** Department of Molecular Biosciences, University of South Florida, 4202 East Fowler Avenue, Tampa, FL 33620, USA; Department of Molecular Biosciences, University of South Florida, 4202 East Fowler Avenue, Tampa, FL 33620, USA; Department of Molecular Biosciences, University of South Florida, 4202 East Fowler Avenue, Tampa, FL 33620, USA; Cancer Biology and Evolution Program, H. Lee Moffitt Cancer Center and Research Institute, 12902 USF Magnolia Drive, Tampa, FL 33612, USA

**Keywords:** Sgs1, Sod2, translesion DNA synthesis, homologous recombination, reactive oxygen species (ROS), mitochondria, chromosome instability

## Abstract

Oxidative stress can damage DNA and thereby contribute to genome instability. To avoid an imbalance or overaccumulation of reactive oxygen species (ROS), cells are equipped with antioxidant enzymes that scavenge excess ROS. Cells lacking the RecQ-family DNA helicase Sgs1, which contributes to homology-dependent DNA break repair and chromosome stability, are known to accumulate ROS, but the origin and consequences of this oxidative stress phenotype are not fully understood. Here, we show that the *sgs1* mutant exhibits elevated mitochondrial superoxide, increased mitochondrial mass, and accumulation of recombinogenic DNA lesions that can be suppressed by antioxidants. Increased mitochondrial mass in the *sgs1*Δ mutant is accompanied by increased mitochondrial branching, which was also inducible in wildtype cells by replication stress. Superoxide dismutase Sod2 genetically interacts with Sgs1 in the suppression of nuclear chromosomal rearrangements under paraquat (PQ)-induced oxidative stress. PQ-induced chromosome rearrangements in the absence of Sod2 are promoted by Rad51 recombinase and the polymerase subunit Pol32. Finally, the dependence of chromosomal rearrangements on the Rev1/Pol ζ mutasome suggests that under oxidative stress successful DNA synthesis during DNA break repair depends on translesion DNA synthesis.

## Teaser

Vidushi, Campos, and Schmidt report that the mitochondrial superoxide dismutase Sod2 suppresses nuclear mutagenesis and chromosome rearrangements under oxidative stress in Saccharomyces cerevisiae. Notably, these chromosome rearrangements not only depend on homologous recombination factors, but also translesion DNA synthesis. Further, the authors identify novel genetic interactions between SOD2 and the RecQ-helicase gene SGS1 in the suppression of chromosome rearrangements and mitochondrial abnormalities. They discuss possible causes and consequences of oxidative stress in cell lacking Sgs1.

## Introduction

Preserving genome integrity is essential for the proper biological functioning of organisms. To this end, eukaryotes have evolved multiple mechanisms of recognizing DNA lesions and repairing them to prevent the accumulation of mutations. One source of genome instability is oxidative stress, which causes major injury to cells by damaging DNA, proteins, and lipids (Carter *et al.* 2005) and is caused by a considerable increase in reactive oxygen species (ROS) (Schrader and Fahimi 2006). ROS include superoxide anions (O_2_·^−^) formed by the reduction of molecular oxygen, hydrogen peroxide (H_2_O_2_) formed by dismutation of O_2_·^−^, and the highly reactive, and, therefore, most toxic, hydroxyl free radicals (OH^−^) that can be formed by decomposition of H_2_O_2_ (Schrader and Fahimi 2006). The interest in ROS stems from its elevated levels in several neurodegenerative pathologies as well as cancer (Barnham *et al.* 2004; Lloret *et al.* 2008). Although still an ongoing debate, ROS has also been implicated as a cause of ageing (Laun *et al.* 2001; Fabrizio *et al.* 2004). Besides being produced by NADPH oxidases and peroxisomes (Salmon *et al.* 2004), endogenous ROS are mainly generated as a natural consequence of aerobic metabolism (Finkel and Holbrook 2000). To prevent ROS from reaching toxic levels cells are naturally equipped to employ antioxidant enzymes such as catalases, superoxide dismutases, and peroxidases to scavenge ROS.

*Saccharomyces cerevisiae* has 2 major superoxide dismutases that are highly conserved and convert superoxide to H_2_O_2_ and oxygen (Fridovich 1974; Miao and Clair 2009). The cytoplasmic Sod1, which also exists in the mitochondrial intermembrane space (Goscin and Fridovich 1972; Sturtz *et al.* 2001), comprises the majority of superoxide dismutase (SOD) activity in yeast (Das *et al.* 2018). The mitochondrial matrix protein Sod2 (Weisiger and Fridovich 1973; Ravindranath and Fridovich 1975; Vögtle *et al.* 2017) promotes chronological life span extension and its loss results in a gradual mitochondrial decline (Longo *et al.* 1996, 1999). Additionally, *SOD2* deletion, like *SOD1* deletion, renders cells hypersensitive to oxygen toxicity (Van Loon *et al.* 1986). H_2_O_2_ can further be reduced by enzymes like Tsa1, which is the most potent scavenger of H_2_O_2_. Belonging to a family of thioredoxin-dependent peroxidases, it reduces H_2_O_2_ and alkyl hydroperoxides by using electrons from NADPH and localizes predominantly to the cytoplasm (Chae *et al.* 1993; Park *et al.* 2000; Irokawa *et al.* 2016).

Some ROS-scavenging enzymes are important for maintaining genome stability. Mutations in *SOD1*, for example, produce an increase in the spontaneous mutation rate (Gralla and Valentine 1991; Huang *et al.* 2003), whereas mutations in *TSA1* have been associated with an increase in spontaneous mutation rate as well as an increase in chromosomal rearrangements (Huang *et al.* 2003; Smith *et al.* 2004). Loss of *SOD1* or *TSA1* also results in increased DNA damage (Ragu *et al.* 2007; Choi *et al.* 2018), underscoring their significant contribution to the maintenance of nuclear genome integrity. Although Sod2 has been shown to prevent mitochondrial genomic instability (Doudican *et al.* 2005), its influence on nuclear genome integrity is unclear. While Sod1 takes up the mantle of being the primary superoxide scavenger and protector of genome stability in yeast, it is Sod2 (MnSOD) that is exceptionally important in higher eukaryotes as *SOD2* disruption is lethal in mice and *Drosophila* (Lebovitz *et al.* 1996; Huang *et al.* 1998; Duttaroy *et al.* 2003), thereby representing the evolutionary selectivity and greater physiological relevance of MnSOD. Moreover, *SOD2* is a putative tumor-suppressor gene and its epigenetic silencing results in cancer cell proliferation (Bravard *et al.* 1992; Li *et al.* 1998).

Oxidative damage to DNA occurs primarily in the form of base modifications and the formation of apurinic/apyrimidinic sites that are mainly repaired by the base excision repair (BER) pathway (Salmon *et al.* 2004). However, recombinational repair of DNA breaks has been associated with the repair of oxidative DNA damage (Slupphaug *et al.* 2003). Sgs1 is a non-replicative RecQ-like DNA helicase in yeast (Sun *et al.* 1999) and a homolog of the human Bloom syndrome (BLM) helicase, the inactivation of which causes a rare genetic disorder called Bloom syndrome (BS), which is characterized by aberrant recombination, chromosome instability, increased predisposition to cancer, short stature, and immunodeficiency (Ellis *et al.* 1995; Mirzaei and Schmidt 2012; Cunniff *et al.* 2017). The role of Sgs1 in homologous-recombination (HR)-mediated double-strand break (DSB) repair, maintenance of genome stability, and repair of stalled or damaged replication forks has been thoroughly investigated (Gangloff *et al.* 1994; Watt *et al.* 1995; Yamagata *et al.* 1998; Kusano *et al.* 1999; Frei and Gasser 2000; Myung *et al.* 2001; Cobb *et al.* 2003; Zhu *et al.* 2008; Cejka *et al.* 2010; Campos-Doerfler *et al.* 2018). In contrast, while mutants lacking Sgs1 are known to accumulate higher ROS than wildtype cells (Ringvoll *et al.* 2007), the cause of elevated ROS, the response of the *sgs1Δ* mutant to elevated ROS, and the contribution of ROS to *sgs1Δ* mutant phenotypes are less well-understood. Reports of an oxidative stress phenotype of human cells lacking BLM (Nicotera *et al.* 1989, 1993; Poot *et al.* 1989; Lloret *et al.* 2008; Subramanian *et al.* 2021) indicate that the role of Sgs1 in mitigating oxidative stress is conserved.

Evidence for a functional interaction between ROS signaling and DSB repair pathways is provided by a genetic study that shows a fitness defect and elevated levels of DNA damage and genome instability in cells lacking both *SOD1* and the recombination factor *RAD51* (Choi *et al.* 2018). Notably, mammalian *SOD1* has been explored as a cancer therapeutic target for the selective killing of cancer cells with defective HR genes, such as *BLM* or *RAD54B* (Sajesh *et al.* 2013; Sajesh and McManus 2015). Moreover, deletion of *RAD51* or *RAD52* in the *tsa1Δ* mutant is synthetically lethal, and this lethality is dependent on the accumulation of ROS-related DNA damage (Huang and Kolodner 2005; Ragu *et al.* 2007). The report of a fitness defect and increased chromosomal rearrangements in the *sgs1*Δ *tsa1*Δ mutant (Huang and Kolodner 2005) highlights the importance of ROS scavenging enzymes in maintaining genome stability in the *sgs1*Δ mutant.

Here, we used SILAC-based quantitative proteomics to evaluate how cells respond to the lack of Sgs1, revealing mitochondrial changes, and investigated the cause and consequences of oxidative stress in the *sgs1*Δ mutant. We report a novel contribution of Sod2 to nuclear genome stability and identify functional interactions between *SOD2* and *SGS1* and other genome maintenance genes, including translesion DNA synthesis (TLS) genes, in the formation of chromosomal rearrangements.

## Materials and methods

### Yeast strains and media

Yeast strains for SILAC labeling, chromatin fractionation, and subsequent mass spectrometry were derived from KHSY5036 (*MATa, ura3-52, trp1Δ63, his3Δ200*). Yeast strains for all other experiments were derived from KHSY802 (*MATa, ura3-52, trp1Δ63, his3Δ200, leu2Δ1, lys2Bgl, hom3-10, ade2Δ1, ade8, hxt13::URA3*). *SGS1* mutant alleles *sgs1-K706A* (*sgs1-hd*) and *sgs1-F1192D* (*sgs1-FD*), and *EXO1* mutant allele *exo1*-E150D/D173A (*exo1-ND*) were previously described (Doerfler and Schmidt 2014; Campos-Doerfler *et al.* 2018). Gene deletions and C-terminal epitope tagging were carried out via recombination-mediated integration of selectable marker cassettes (Longtine *et al.* 1998) at the chromosomal loci by LiAc-mediated transformation (Gietz and Woods 2006). Haploids with multiple mutations were obtained by sporulating diploid heterozygous for the desired mutations and selection by random spore analysis on selective media or by PCR as previously described (Rockmill *et al.* 1991; Mirzaei *et al.* 2011). Yeast was grown at 30° in yeast extract (10 g/L), peptone (20 g/L), and dextrose (20 g/L) (YPD) or synthetic complete media with or without agar (20 g/l). All yeast strains used in this study are listed in Supplementary Table 1 and are available upon request.

### Spot assay

Sensitivity of exponentially growing cell cultures to DNA damaging and oxidizing agents was examined with spot assays. Cultures from single colonies were grown overnight at 30° in liquid YPD, adjusted to an OD_600_ of 0.2, and grown to an OD_600_ of 0.5, followed by spotting 10-fold serial dilutions on YPD and YPD containing indicated concentrations of paraquat (PQ; Sigma), hydrogen peroxide (H_2_O_2_; Santa Cruz Biotechnology), hydroxyurea (HU; US Biological), and methyl methanesulfonate (MMS; Acros Organics). Growth was monitored at 30° and images were acquired every 12–24 hours for 2–4 days with a GelDoc IT Imaging system.

### Chromatin enrichment for SILAC-based mass spectrometry

Isotope labeling of arginine and lysine was carried out according to (Syed *et al.* 2016). Briefly, KHSY5144 (*lys2Δ arg4Δ*) was grown at 30° in “light” medium containing 15 mg/L L-arginine and 30 mg/L L-lysine whereas KHSY5226 (*lys2Δ arg4Δ sgs1Δ*) was grown in “heavy” medium containing 15 mg/L L-arginine (^13^C_6_) and 30 mg/L L-lysine (^13^C_6_). Chromatin isolation was carried out as previously described (Kubota *et al.* 2012; Syed *et al.* 2016). Briefly, cells were suspended in pre-spheroplast buffer (100 mM PIPES/KOH, pH 9.4, 10 mM dithiothreitol (DTT), 0.1% sodium azide), followed by a 60-minute incubation in spheroplast buffer (50 mM KH_2_PO_4_/K_2_HPO_4_, pH 7.4; 0.6 M sorbitol, 10 mM DTT) containing Zymolase-100 T. Spheroplasts were washed, resuspended in wash buffer (20 mM KH_2_PO_4_/K_2_HPO_4_, pH 6.5; 0.6 M sorbitol, 1 mM MgCl_2_, 1 mM DTT, 20 mM β-glycerophosphate, 1 mM phenylmethylsulfonyl fluoride (PMSF)), and overlaid onto 7.5% Ficoll-sorbitol cushion buffer (7.5% Ficoll, 20 mM KH_2_PO_4_/K_2_HPO_4_, pH 6.5; 0.6 M sorbitol, 1 mM MgCl_2_, 1 mM DTT, 20 mM β-glycerophosphate, 1 mM PMSF, Protease inhibitor cocktail (EDTA free, Thermo Scientific)), followed by centrifugation. Spheroplasts were resuspended and dropped onto 18% Ficoll, followed by homogenization and removal of unbroken cells by centrifugation at 5000×g for 10 minutes twice. Nuclei were pelleted and cytoplasmic fraction was collected, followed by nuclear lysis with 0.25% Triton X-100. Lysate was overlaid onto a buffer containing 30% sucrose, centrifuged, and the chromatin pellet collected.

### Sample preparation and LC-MS/MS

Chromatin pellets were prepared for mass spectrometry using filter-aided sample preparation (FASP) as previously described (Syed *et al.* 2016). Briefly, proteins were alkylated with iodoacetamide (IAA), buffer exchanged with urea followed by ammonium bicarbonate, and finally digested with Trypsin/Lys-C overnight at 37°. Peptides were eluted and subsequently desalted using C18 solid-phase extraction cartridges (Waters) with a vacuum manifold. Desalted peptides were lyophilized in a vacuum concentrator. Peptides were resuspended in 0.1% formic acid for liquid chromatography (LC)-MS/MS analysis. Peptides were separated using a 75 µm × 50 cm C18 reversed-phase-HPLC column (Thermo Scientific) on an Ultimate 3000 UHPLC (Thermo Scientific) with a 120-minute gradient (2–32% acetonitrile with 0.1% formic acid) and analyzed on a hybrid quadrupole-Orbitrap instrument (Q Exactive Plus, Thermo Fisher Scientific). Full MS survey scans were acquired at 70,000 resolution and the top 10 most abundant ions were selected for MS/MS analysis. Raw data files were processed in MaxQuant (Cox and Mann 2008) and searched against the *Saccharomyces* genome database (SGD). Search parameters included constant modification of cysteine by carbamidomethylation and the variable modification of methionine oxidation. Proteins were identified using the filtering criteria of 1% protein/peptide false discovery rate (Hochberg and Benjamini 1990; Cox and Mann 2008). SILAC (H/L) ratios obtained for each replicate (*n* = 3) were analyzed by an outlier test (SigA test) in Perseus (Tyanova *et al.* 2016). Our focus for this analysis was statistical significance related to magnitude fold-change (SigA test, *P* < 0.05); however, we also addressed variance across replicates through additional filtering. Specifically, proteins were only included in the dataset if at least 2 of the 3 replicates had a measured stable isotope labeling by amino acids in cell culture (SILAC) ratio (Chaput *et al.* 2016). Additionally, proteins were only included in the final dataset if a coefficient of variation (CV) of less than 30% related to measured SILAC ratios across replicates was achieved. The mass spectrometry proteomics data have been deposited to the ProteomeXchange Consortium via the PRIDE (Perez-Riverol *et al.* 2022) partner repository with the dataset identifier PXD040745.

### Protein extraction and western blot analysis

Whole-cell, cytoplasmic, nucleoplasm, and chromatin extracts from yeast expressing Myc-tagged Sod2 in the absence or presence of Sgs1 were prepared as previously described (Syed *et al.* 2016). Samples were separated on 10% SDS-PAGE, transferred to PVDF membrane, and incubated with c-myc antibody (Santa Cruz Biotechnology) to detect myc epitope-tagged Sod2, with histone H3 (Abcam) and Adh1 (Abcam) antibodies to verify chromatin enrichment, and with α-tubulin (Santa Cruz Biotechnology) and replication factor A (Agrisera) antibodies as loading controls.

### Fluctuation assay

Cells with gross-chromosomal rearrangements (GCRs) were identified by their resistance to both canavanine (can^r^) and 5-fluoro-orotic acid (5-FOA^r^) due to simultaneous inactivation of the *CAN1* and *URA3* genes, which are present within the nonessential end of the left arm of chromosome V (Schmidt *et al.* 2006a). Ten-milliliter cultures from single colonies were grown for 2 days at 30° with or without PQ, followed by plating appropriate dilutions on YPD to obtain the viable cell count and plating the remaining culture on synthetic media lacking arginine and uracil, supplemented with 60 mg/L canavanine (Sigma) and 1 g/L 5-FOA (US Biological) to select for cells with GCRs after incubation for 4 days at 30°. The rate of accumulating GCRs was determined by fluctuation analysis, and the median GCR rate from 15 cultures from 3 isolates per genotype was reported with 95% confidence intervals (Nair 1940; Lea and Coulson 1949; Schmidt *et al.* 2006a). The rate of accumulating mutations at the *CAN1* locus was determined by fluctuation analysis by the method of the median (Nair 1940; Lea and Coulson 1949; Reenan and Kolodner 1992). Briefly, 6-ml cultures were grown for 2 days at 30° in liquid YPD with or without PQ and aliquots plated on synthetic media lacking arginine and supplemented with 60 µg/mL canavanine while appropriate dilutions were plated on YPD to obtain viable cell count. Fifteen cultures from 3 isolates per genotype were analyzed. The *CAN1* ORF was amplified from can^r^ clones by PCR and PCR products were analyzed by agarose gel electrophoresis.

### Determination of ROS and mitochondrial superoxide content

Intracellular ROS levels were measured with 2′,7′-dichlorodihydrofluorescein (DCFH; λ_exc_ 485 nm λ_em_ 538 nm; Sigma) while mitochondrial superoxide levels were determined using the dihydroethidium (DHE) derivative MitoSOX Red (Invitrogen; λ_exc_ 488–510 nm λ_em_ 580 nm) (Johnson-Cadwell *et al.* 2007; Quaranta *et al.* 2011; Ramirez *et al.* 2014). Exponentially growing cells were stained with 10 µM DCFH or 5 µM MitoSOX Red for 30 minutes at 30° with gentle rocking as described (Ringvoll *et al.* 2007; Sariki *et al.* 2016), followed by washing and resuspension in PBS, and single-cell analysis of 100,000 cells by flow cytometry. Data was analyzed using Becton Dickinson (BD) FACSDiva software and is presented as the average of the median fluorescence values from 3 biological replicates, after adjusting for autofluorescence from unstained cells.

### Determination of mitochondrial mass

Mitochondrial mass was determined using nonyl acridine orange (NAO; λ_ex_ = 490 nm, λ_em_ = 518 nm; Invitrogen), which binds to cardiolipin independently of the energetic status of mitochondria (Benel *et al.* 1989). Exponentially growing cells were incubated in the dark with 1 µg/mL NAO for 20 minutes at 30°, followed by washing, resuspension in PBS, and flow cytometry of 100,000 cells (Lai *et al.* 2002).

### Fluorescence microscopy

To assess accumulation of recombinogenic DNA lesions in yeast nuclei, the recombination factor Rad52 was tagged with GFP, cell cultures grown to an OD_600_ of 0.5–0.6, and the percentage of cells with Rad52-GFP foci determined by fluorescence microscopy (Lisby *et al.* 2001) on a BZ-X710 (Keyence); cells were cultured in the presence of 0.5 mM H_2_O_2_ for 30 mins or varying concentrations of *N*-acetyl-L cysteine (NAC) for 2 hours. To assess mitochondrial morphology, yeast expressing GFP-tagged Aco1 (Klinger *et al.* 2010) were grown to an OD_600_ of 0.8 in YPD before preparing cells for microscopy or incubated in 0.03 mM PQ for 18 hours, followed by dilution to OD_600_ of 0.2 and culturing to an OD_600_ of 0.8 in 0.03 mM PQ, followed by fluorescence microscopy on a BZ-X170 (Keyence) with a 60 × oil immersion objective. For visualizing mitochondrial morphology under replication stress, cells were grown to an OD_600_ of 0.5 and treated with 10 mM or 200 mM HU (US Biological) for 3 hours, and images were acquired on a BZ-X170 fluorescence microscope (Keyence). To assess the intracellular localization of red-fluorescent-protein-tagged Sod2 (Sod2-RFP), cells were grown for fluorescence microscopy to OD_600_ of 0.8 in the absence of PQ or 1 mM PQ for 1 hour. For 24-hour PQ treatment, cells were grown in 1 mM PQ for 24 hours, diluted to an OD_600_ of 0.2, and cultured to an OD_600_ of 0.8 in 1 mM PQ before harvesting cells for microscopy. Cells were fixed in 3.7% formaldehyde for 1 hour and stained with 50 ng/mL 4′,6-diamidino-2-phenylindole in an antifade mounting medium (DAPI; Vector Laboratories) to visualize nuclei. Following washes with PBS, cell suspensions were mounted on agarose pads over glass slides and imaged using a BZ-X170 fluorescence microscope (Keyence) with a 60 × oil immersion objective. One DIC image and 10–20 fluorescent images at 0.3 µm intervals along the z-axis were acquired to allow inspection of all focal planes. At least 200–250 cells were scored for Rad52-GFP foci and Sod2-RFP localization and 100 cells for mitochondrial morphology. Data is presented as the average of 3 biological replicates per genotype.

## Results

### Evaluation of the cellular response to *SGS1* deletion by SILAC-based mass spectrometry

**Fig. 1.**
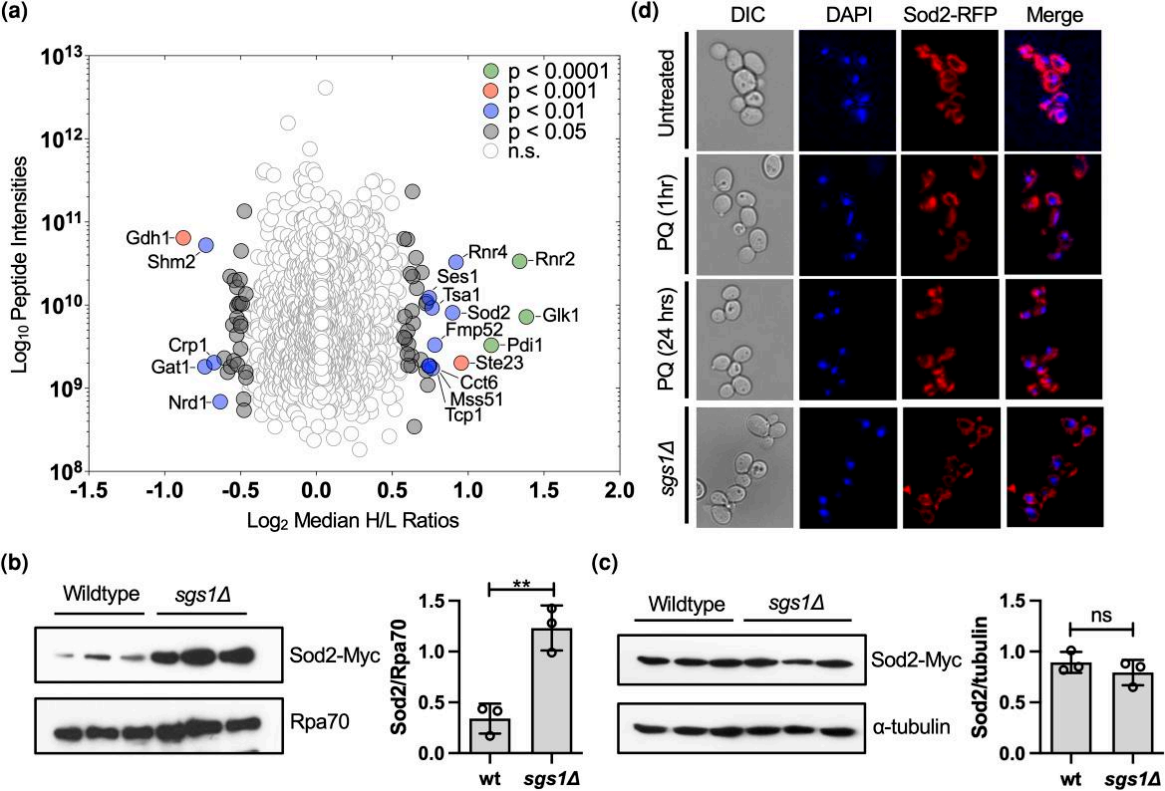
Cellular response to *SGS1* deletion. a) SILAC-based quantitative proteomic analysis comparing chromatin-enriched fractions of wild-type cells and the *sgs1Δ* mutant. SILAC analysis was performed in triplicate with the median ratios (H/L) of heavy-labeled (*sgs1*Δ) to light-labeled (wildtype) proteins relative to protein abundance (estimated by summed peptide intensities) represented in this plot. The overall median ratios obtained from the entire dataset were used to demonstrate statistical significance based on magnitude fold-change (SigA test, *P* < 0.05). Details of additional statistical filtering are provided in Materials and Methods section. For a list of all proteins that significantly changed in the *sgs1*Δ mutant, see Supplementary Table 2 b) Western blot and quantification of Sod2-myc levels in 3 independent chromatin-enriched fractions from wild-type cells and the *sgs1Δ* mutant. Rpa70 was used as a loading control. The ratio between Sod2-myc and RPA70 is reported with standard deviation. c) Western blot and quantification of Sod2-myc expression levels in 3 independent whole-cell extracts from wild-type cells and the *sgs1Δ* mutant. α-tubulin was used as a loading control. The ratio between Sod2 and tubulin is reported with a standard deviation. d) Fluorescence microscopy images of RFP-tagged Sod2 and DAPI staining in wildtype cells in the absence and presence of PQ and in the absence of Sgs1 (*sgs1*Δ). The statistical significance of differences in b) and c) was determined with a Student's *t*-test and reported as **P ≤ 0.01; ns, not significant.

Cells lacking the RecQ family helicase Sgs1 exhibit DNA recombination and replication defects (Frei and Gasser 2000; Schmidt and Kolodner 2004; Branzei *et al.* 2006; Mankouri *et al.* 2007; Mimitou and Symington 2010; Nielsen *et al.* 2013; Fasching *et al.* 2015). To better understand the cellular response to the absence of Sgs1 and identify novel functional interactors of Sgs1, we performed SILAC-based quantitative mass spectrometry of chromatin-enriched fractions isolated from a mixture of light-labeled wildtype cells and heavy-labeled cells with an *SGS1* deletion (*sgs1Δ*) (Fig. 1a, Supplementary Fig. 1). We identified 68 proteins that were significantly changed in the chromatin-enriched fraction of the *sgs1*Δ mutant (Fig. 1a, Supplementary Table 2). We were particularly interested in the proteins whose levels increased as they may include new genetic interactions with the *sgs1Δ* mutation. Notable increases were observed for Rnr2 and Rnr4, subunits of ribonucleotide reductase, which catalyzes balanced dNTP production throughout the cell cycle and during DNA damage (Elledge *et al.* 1993), and could be a response to elevated DNA lesions and genome instability in the *sgs1Δ* mutant (Myung *et al.* 2001; Chang *et al.* 2005). The ATP-dependent RNA helicase Nam7/Upf1, which functions in nonsense-mediated mRNA decay but is also required for telomere length maintenance (Leeds *et al.* 1992; Lew *et al.* 1998; Gatbonton *et al.* 2006), was also significantly upregulated (*P* < 0.05); its human homolog UPF1 also helps preserve telomere stability and is a potential tumor suppressor (Azzalin *et al.* 2007; Chen *et al.* 2021). Sgs1's functions in telomere maintenance involving telomeric end processing and telomere length maintenance (Bryan *et al.* 1997; Cohen and Sinclair 2001; Johnson *et al.* 2001; Cesare and Reddel 2008; Bonetti *et al.* 2009) raise the possibility of a functional interaction with Nam7/Upf1. Interestingly, GO Slim Term Mapping of the upregulated proteins revealed mitochondrial proteins as the largest fraction (13/37 mapped proteins; Supplementary Fig. 1c and Supplementary Table 2), including the superoxide dismutase Sod2 (*P* < 0.01), which eliminates superoxide radicals in mitochondria (Whittaker and Whittaker 2012). Although increased ROS has been detected in cells lacking Sgs1, and *SGS1* was identified in a screen for genes important for the survival and chromosome stability of cells lacking the cytoplasmic peroxiredoxin Tsa1 (Huang and Kolodner 2005; Ringvoll *et al.* 2007), the oxidative stress phenotype in the *sgs1*Δ mutant and the link between oxidative stress and chromosome instability are poorly understood. Therefore, this study focused on understanding the importance of the mitochondrial antioxidant Sod2 in maintaining nuclear chromosome stability and its functional interaction with Sgs1.

First, we verified Sod2 expression levels in the *sgs1*Δ mutant by Western blotting. This confirmed enrichment of Sod2 in the chromatin fraction of the *sgs1Δ* mutant (Fig. 1b, Supplementary Fig. 1b) but revealed no change in Sod2 levels in whole cell extracts (Fig. 1c), indicating that Sod2 enrichment in the chromatin fraction was not due to increased Sod2 expression in the *sgs1Δ* mutant. Therefore, we considered the possibility that the increase of Sod2 in the chromatin-enriched fraction of the *sgs1*Δ mutant was due to relocalization of Sod2 from the mitochondria to the nucleus as had previously been shown for Sod1 in cells under oxidative stress where Sod1 demonstrated cytoplasmic-to-nuclear relocalization (Tsang *et al.* 2014). Using RFP-tagged Sod2, we confirmed that Sod2-RFP was distributed throughout mitochondria (Vögtle *et al.* 2017) but did not colocalize with DAPI-stained nuclei during PQ-induced oxidative stress or in the *sgs1Δ* mutant (Fig. 1d). Only a small fraction of cells (1.6% ± 0.5) did show some overlap between Sod2-RFP and DAPI stained nuclei (Supplementary Fig. 2) whereas Sod1 localized to the nucleus in the majority of oxidatively stressed cells (Tsang *et al.* 2014). Thus, the enrichment of Sod2 in the chromatin fraction of the *sgs1*Δ mutant could not be explained by Sod2 relocalization to the nucleus. Considering the enrichment of several other mitochondrial proteins in the chromatin fraction of the *sgs1*Δ mutant besides Sod2 (Supplementary Table 2) and co-purification of mitochondrial DNA-associated proteins with nuclear chromatin (Tan *et al.* 2007; Kim *et al.* 2011; Cuevas-Bermúdez *et al.* 2019) we considered that mitochondrial network changes in the *sgs1*Δ mutant could explain the elevated levels of Sod2 and other mitochondrial proteins in the chromatin fraction of the *sgs1*Δ mutant. Therefore, we next evaluated mitochondrial morphology in the *sgs1*Δ mutant.

### Absence of Sgs1 or HU-induced replication stress induces branched mitochondrial morphology

**Fig. 2.**
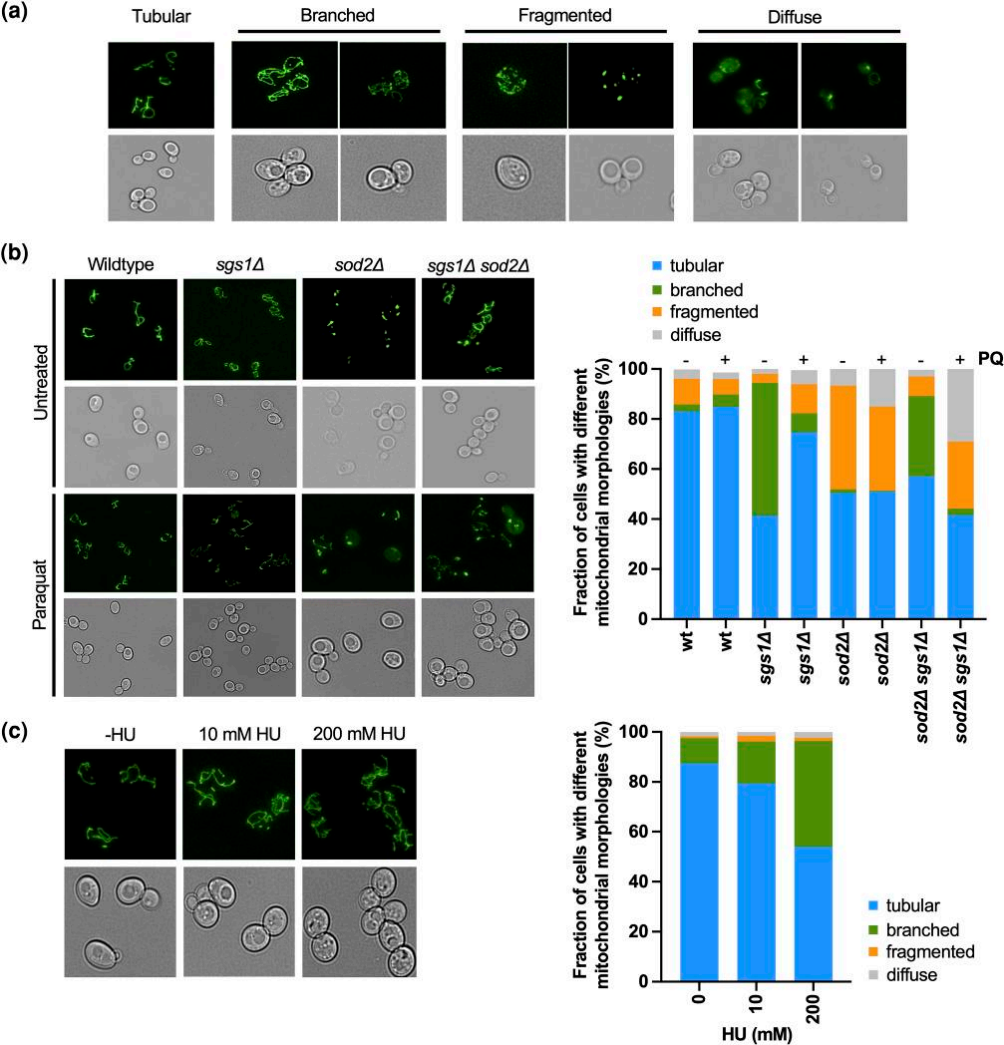
Changes in mitochondrial morphology upon deletion of *SGS1* and/or *SOD2* in the presence or absence of PQ. a) Overview of the 4 major mitochondrial morphologies (tubular, branched, fragmented, diffuse) we observed in wildtype cells or the *sgs1*Δ and *sod2*Δ mutants in the presence or absence of PQ (0.03 mM). Mitochondria were visualized by tagging mitochondrial aconitase Aco1-GFP, followed by fluorescence microscopy. b) Left panel: Representative images of different mitochondrial morphologies in wildtype and *sgs1*Δ and *sod2*Δ mutants in the presence and absence of PQ (0.03 mM). Mitochondria were visualized by GFP-tagging Aco1, followed by fluorescence microscopy. Right panel: The fraction of cells with either tubular, branched, fragmented, or diffuse mitochondrial morphology was determined from 3 experiments and a minimum of 100 cells of each strain and treatment condition. The mean is presented; for mean ± SD see Supplementary Table 3. c) Left panel: Representative images of mitochondrial morphology in wildtype cells treated with a low dose (10 mM) or high dose (200 mM) of HU. Mitochondria were visualized by GFP-tagging Aco1, followed by fluorescence microscopy. Right panel: The fraction of cells with either tubular, branched, fragmented, or diffuse mitochondrial morphology was determined from a minimum of 100 cells from 3 experiments performed in the absence of HU and for each HU concentration. The mean is presented; for mean ± SD see Supplementary Table 4.

Mitochondria provide cells with energy via oxidative phosphorylation and are the predominant source of cellular ROS. The mitochondria in wildtype yeast cells appear as long, continuous tubules, which are the result of balanced fission and fusion dictated, among others, by metabolic requirements and life cycle events (Shaw and Nunnari 2002; Jakobs *et al.* 2003). Continuous tubules and branched tubules are associated with functional mitochondria whereas fragmented morphology indicates mitochondrial dysfunction (Aerts *et al.* 2009; Rudan *et al.* 2018). To characterize morphological states of mitochondria in cells lacking Sgs1 and/or Sod2, we tagged the mitochondrial matrix marker protein Aco1 with GFP (Aco1-GFP) and observed the cells by fluorescence microscopy, revealing 4 distinct morphological states that were easily scorable: long tubules, branched tubules, fragmented mitochondria, and diffuse mitochondria (Fig. 2a). Based on these 4 categories, we categorized mitochondrial morphology in at least 100–200 cells per genotype in the absence of exogenous stress and during PQ-induced oxidative stress (Fig. 2, b and c, Supplementary Table 3). Surprisingly, the *sgs1*Δ mutant displayed a large fraction of branched mitochondria (47%±1.7%) signifying a change from the continuous tubules that are characteristic of wildtype cells (82%±3.2). In contrast, many mitochondria in the *sod2*Δ mutant were fragmented (41%±5.8), indicative of dysfunctional mitochondria. When we introduced the *sgs1Δ* mutation into the *sod2*Δ mutant the branched mitochondrial morphology (32%±5.8), characteristic of the *sgs1*Δ mutant, dominated over the fragmented morphology (8%±1.7), characteristic of the *sod2*Δ mutant, indicating that mitochondrial morphology in the *sgs1Δ sod2Δ* mutant was more similar to the *sgs1*Δ mutant than the *sod2*Δ mutant. Although cell cycle analysis revealed a slight increase in the fraction of *sod2Δ sgs1Δ* cells in G2/M compared to the single mutants, mitochondrial branching was not limited to a particular cell cycle phase (Supplementary Fig. 3), suggesting that the small change in cell cycle distribution is unlikely to be responsible for the observed changes in mitochondrial morphology.

Based on previous studies (Rudan *et al.* 2018), our findings of increased ROS and branched mitochondrial morphology suggest that the mitochondria in the *sgs1Δ* mutant are functional. Moreover, chronological and replicative lifespans of the *sgs1Δ* mutant growing on glycerol, a non-fermentable carbon source, are similar to those of wildtype cells (Ringvoll *et al.* 2007), further ruling out mitochondrial dysfunction in the *sgs1Δ* mutant. Furthermore, since there was no difference in mitochondrial morphology between the *sgs1Δ* mutant growing on 0.5 and 2% glucose (Supplementary Fig. 4), the observed branching appears to be independent of respiratory activity. Notably, there is evidence that high energy demands in a cell can result from increased DNA damage, which requires more ATP production to help repair DNA (Gafter-Gvili *et al.* 2011; Kulkarni *et al.* 2011). Since mitochondrial branching indicates increased mitochondrial activity (Rudan *et al.* 2018), we suspected that the increase in mitochondrial branching in the *sgs1Δ* mutant could be due to an increased need for ATP to respond to DNA lesions. Indeed, upon exposure to HU, which induces replication stress, wildtype cells underwent increased mitochondrial branching in a dose-dependent manner (Fig. 2c, Supplementary Table 4), providing evidence that the cellular response to replication stress is a cause for mitochondrial branching.

After treatment with PQ, which induces mitochondria-dependent superoxide production, the mitochondrial morphology in wildtype cells did not undergo a major change (Fig. 2b). In the *sgs1*Δ mutant, cells with branched mitochondria decreased sharply while those with tubular morphology increased, further supporting that the branched morphology in the *sgs1*Δ mutant is not caused by the increased ROS characteristic of *sgs1*Δ mutants but is due to DNA lesions and replication stress caused by the absence of Sgs1. Since branched and tubular mitochondria are considered functional this change in their proportions likely did not affect mitochondrial function in the *sgs1*Δ mutant. Fragmented or diffuse mitochondria, indicative of mitochondrial dysfunction, were still only seen in a small fraction of *sgs1*Δ cells (fragmented: 3.6% ± 3.4 vs 11.7% ± 4.9 with PQ, *P* = 0.079; diffuse: 1.9% ± 0.86 vs 5.6% ± 3 with PQ, *P* = 0.11), indicating that the vast majority of *sgs1*Δ cells in the presence or absence of induced oxidative stress had functional mitochondria. In the *sod2*Δ mutant, PQ exposure did not change the fraction of cells with functional mitochondria and only caused a small increase in cells with diffuse mitochondria (6.6% ± 3.5 to 14.9% ± 3.5; *P* < 0.05), indicative of a mild increase in dysfunction or cell death. Fragmented and diffuse mitochondrial morphologies spiked, however, in the *sgs1Δ sod2Δ* mutant after PQ exposure (fragmented: 7.9% ± 1.7 to 26.8% ± 3.9; *P* < 0.01; diffuse: 2.5% ± 0.9 to 29% ± 3.2, *P* < 0.001), raising the possibility of a negative genetic interaction between *sgs1Δ* and *sod2Δ*.

### Causes of the oxidative stress phenotype of the *sgs1*Δ mutant

**Fig. 3.**
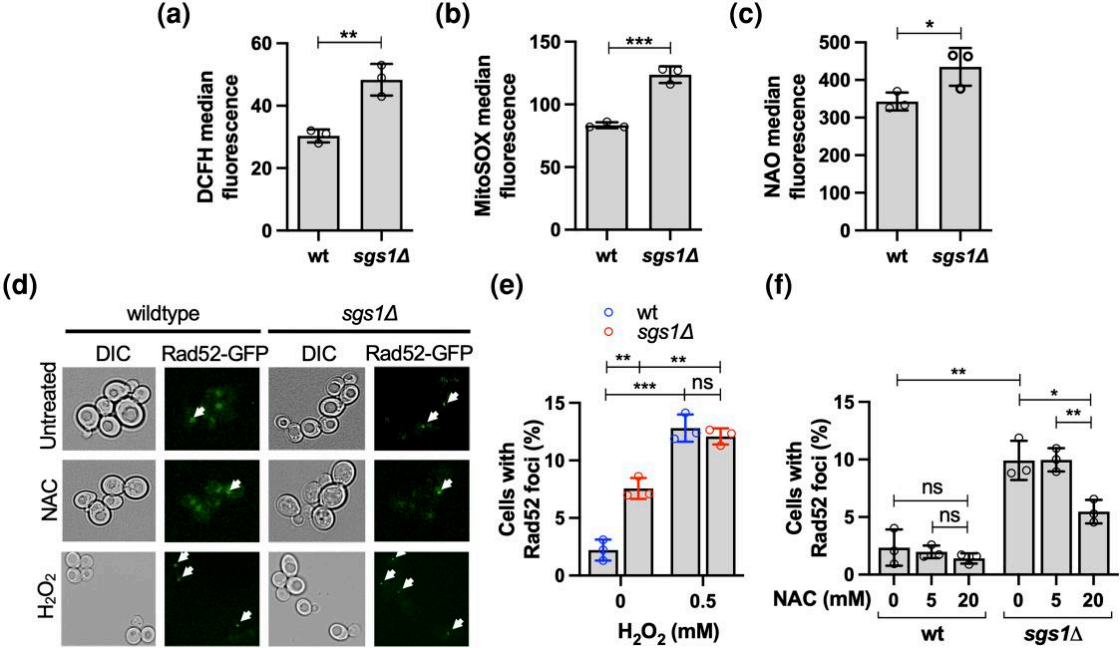
Endogenous ROS, mitochondrial mass, and recombinogenic DNA lesion formation in the *sgs1*Δ mutant. a) Measurement of ROS in the *sgs1*Δ mutant by flow cytometry using the fluorescent dye DCFH diacetate. b) Measurement of mitochondrial superoxide content in the *sgs1*Δ mutant by flow cytometry using MitoSOX Red fluorescent dye. c) Measurement of mitochondrial mass in the *sgs1*Δ mutant by flow cytometry using the fluorescent dye NAO. d) To detect recombinogenic DNA lesions, homologous recombination factor Rad52 was tagged with GFP (Rad52-GFP), and foci formation detected by fluorescence microscopy (BZ-X170, Keyence) of exponentially growing cell cultures either untreated or treated with the antioxidant NAC (20 mM) for 2 hours or H_2_O_2_ (0.5 mM) for 30 minutes. Representative images are shown with arrows pointing to nuclei with Rad52-GFP foci. In nuclei without Rad52-GFP foci, the GFP signal appears diffused throughout the nucleus. e) Percentage of cells with Rad52-GFP foci in cultures of wildtype cells and the *sgs1*Δ mutant in the presence or absence of oxidative stress induced by H_2_O_2_. Exponentially growing cell cultures were incubated at 0.5 mM H_2_O_2_ for 30 minutes and imaged by fluorescence microscopy (BZ-X170, Keyence). f) Effect of the antioxidant NAC (5 mM, 20 mM) on the percentage of cells with Rad52-GFP foci in cultures of wildtype cells and the *sgs1*Δ mutant. Exponentially growing cell cultures were incubated at the indicated concentration of NAC for 2 hours and imaged by fluorescence microscopy (BZ-X170, Keyence). Experiments were performed in triplicate and the mean ± SD is reported. Statistical significance of differences was determined with a Student's *t*-test and reported as *P < 0.05; **P ≤ 0.01; ***P ≤ 0.001; ns, not significant.

To better understand the oxidative stress phenotype of the *sgs1Δ* mutant (Ringvoll *et al.* 2007), we measured endogenous ROS levels by DCFH staining and MitoSox staining and found a 40% increase in overall ROS levels (Fig. 3a) and a 50% increase in mitochondrial superoxide content (Fig. 3b) in the *sgs1Δ* mutant. Since mitochondria are the predominant sources of cellular ROS (Rossignol and Frye 2012), we asked if this increase in ROS in the *sgs1*Δ mutant was due to an increase in mitochondrial mass. Performing flow cytometry using NAO, which stains mitochondria independently of their energetic status (Benel *et al.* 1989; Lai *et al.* 2002), we found a significant 26% increase in mitochondrial mass in the *sgs1Δ* mutant (Fig. 3c). Taken together with the increased mitochondrial branching in the *sgs1Δ* mutant (Fig. 2b) and the inducibility of mitochondrial branching by replication stress (Fig. 2c), these results suggest that the oxidative stress phenotype of the *sgs1Δ* mutant could be due to genome instability that induces increased mitochondrial mass and branching, which in turn causes increases in cellular ROS.

To determine the effect of increased ROS in the *sgs1Δ* mutant on nuclear DNA integrity, we measured the percentage of cells that exhibited foci of the nuclear homologous recombination factor Rad52. Using fluorescence microscopy, we observed more spontaneous Rad52-GFP foci in the *sgs1Δ* mutant than in the wildtype (Fig. 3, d and e), consistent with previous reports (Chang *et al.* 2005). Upon treatment with H_2_O_2_, however, Rad52 foci increased 6-fold in wildtype cells, but only 1.7-fold in the *sgs1Δ* mutant (Fig. 3, d and e), raising the possibility that endogenous oxidative stress caused by the absence of Sgs1 is a source of Rad52-GFP foci. To test this hypothesis, we treated the *sgs1*Δ mutant with varying amounts of the antioxidant NAC, a well-established ROS scavenger that reacts with H_2_O_2_ and hydroxyl radicals (Aruoma *et al.* 1989; Zafarullah *et al.* 2003; Kim *et al.* 2013). Indeed, NAC supplementation resulted in a nearly 50% reduction of *sgs1Δ* cells with Rad52-GFP foci, but had no significant effect on spontaneous Rad52-GFP foci formation in wildtype cells (Fig. 3, d and f), suggesting that the increase in endogenous ROS significantly contributes to the formation of recombinogenic DNA lesions in cells lacking Sgs1.

### Sod2 suppresses nuclear genome instability under oxidative stress and genetically interacts with Sgs1

**Fig. 4.**
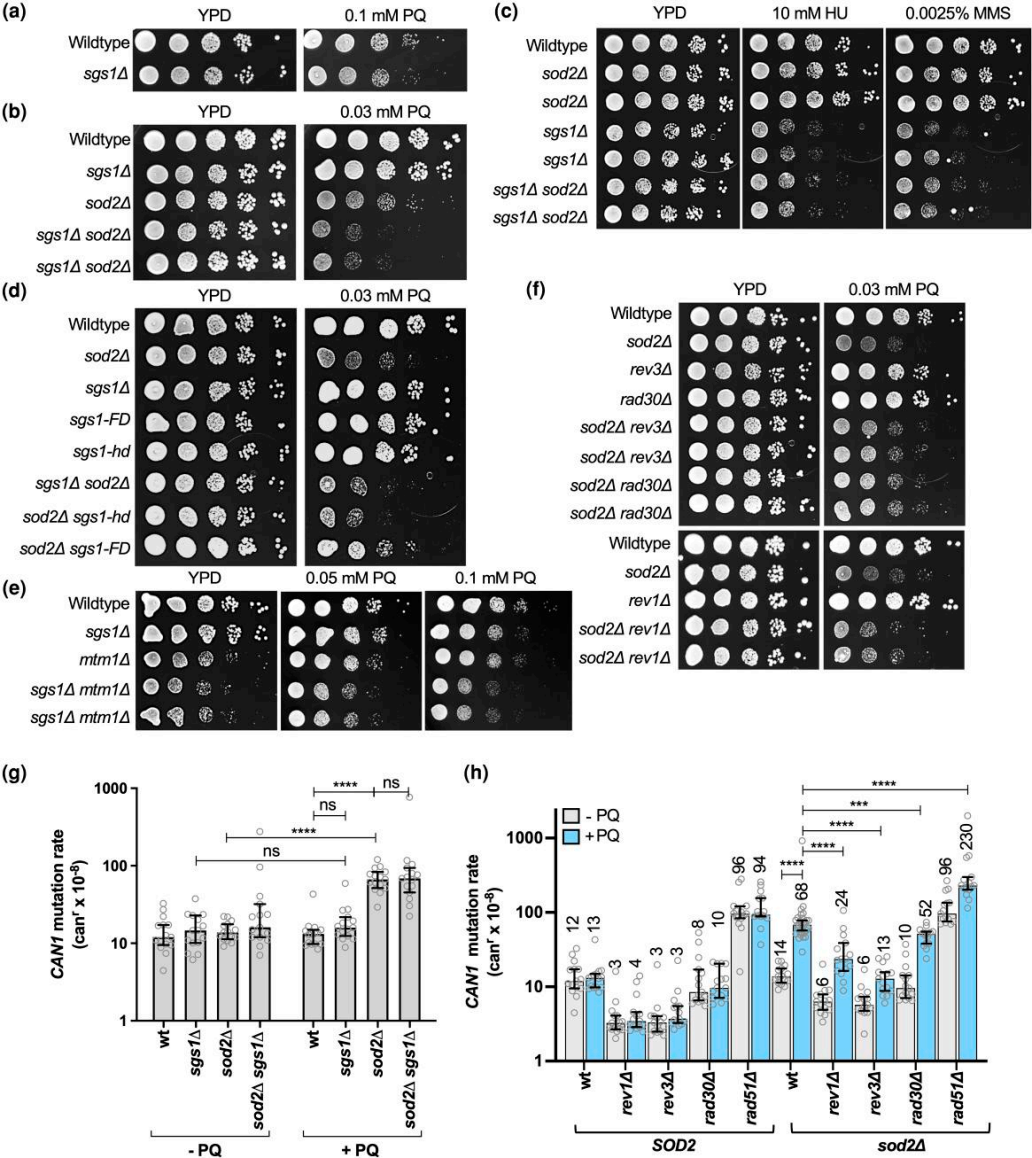
Sensitivity to DNA damage, replication stress, oxidative stress, and level of genome instability of cells lacking Sgs1 and Sod2 activities. a) Spot assay to determine the sensitivity of the *sgs1*Δ mutant to PQ. Ten-fold-dilutions of exponentially growing cell cultures were spotted on YPD or on YPD supplemented with 0.1 mM PQ and incubated at 30°. b) Spot assay to determine the sensitivity of the *sgs1*Δ *sod2*Δ mutant to PQ. Ten-fold-dilutions of exponentially growing cell cultures were spotted on YPD or YPD supplemented with 0.03 mM PQ and incubated at 30°. c) Spot assay to determine the sensitivity of the *sgs1*Δ *sod2*Δ mutant to HU and MMS. Ten-fold-dilutions of exponentially growing cell cultures were spotted on YPD or YPD supplemented with the indicated concentrations of HU or MMS and incubated at 30°. d) Spot assay to determine PQ-sensitivity of the *sod2*Δ mutant harboring *sgs1* mutations that interrupt helicase activity (*sgs1-hd*) or Rad51-binding (*sgs1-FD*). Ten-fold-dilutions of exponentially growing cell cultures were spotted on YPD or YPD supplemented with 0.03 mM PQ and incubated at 30°. e) Spot assay to determine PQ-sensitivity of the *sgs1*Δ mutant with a deletion of *MTM1*. Ten-fold-dilutions of exponentially growing cell cultures were spotted on YPD or on YPD supplemented with 0.1 mM or 0.05 mM PQ and incubated at 30°. f) Spot assay to determine the effect of *rev1Δ*, *rev3*Δ, or *rad30*Δ mutations on PQ sensitivity of the *sod2*Δ mutant. Ten-fold-dilutions of exponentially growing cell cultures were spotted on YPD or YPD supplemented with 0.03 mM PQ and incubated at 30°. g) Rates of accumulating inactivating mutations in *CAN1* in cells harboring *sgs1*Δ and/or *sod2*Δ mutations in the presence or absence of PQ. Rates of accumulating canavanine-resistant (can^r^) clones were determined from at least 15 cell cultures (gray dots) for each yeast strain in the presence or absence of 0.03 mM PQ. The statistical significance of differences was determined by a Mann–Whitney test and reported as **** *P* ≤ 0.0001; ns, not significant. Numbers above the columns in the graph indicate the median rate of accumulating mutations conferring resistance to canavanine (per cell/generation, can^r^×10^−8^). For a complete list of all can^r^ rates with 95% confidence intervals see also Supplementary Table 5. h) Rates of accumulating inactivating mutations in the *CAN1* gene (can^r^) of *sod2*Δ mutants harboring deletions of TLS genes (*rev1Δ, rev3Δ, rad30Δ*) or a homologous recombination gene (*rad51*Δ) in the presence or absence of PQ. At least 15 cell cultures (gray dots) from 3 isolates for each yeast strain were analyzed. The median rate with 95% confidence intervals is shown. Numbers above the columns in the graph indicate the median rate of accumulating mutations conferring resistance to canavanine (per cell/generation, can^r^×10^−8^). For a complete list of all can^r^ rates with 95% confidence intervals see Supplementary Table 5. Statistical significance of differences was determined by a Mann–Whitney U-test and reported as ***P ≤ 0.001; ****P ≤ 0.0001.

To better understand ROS as a source of nuclear genome instability we investigated the effect of the lack of a mitochondrial antioxidant on the formation of small-scale mutations (*CAN1* mutation assay) and GCR assay in the *sod2Δ* mutant and a possible genetic interaction with the *sgs1*Δ mutation, which caused mild sensitivity to PQ (Fig. 4a). Notably, we observed a synergistic increase in sensitivity of the *sod2*Δ *sgs1*Δ mutant to PQ but not to HU and MMS, which result in replication stress and DNA damage (Fig. 4, b and c), suggesting a PQ-specific functional interaction between *SGS1* and *SOD2*. This functional interaction is dependent on Sgs1 helicase activity since the *sod2Δ sgs1-hd* mutant was as sensitive to PQ as the *sgs1Δ sod2Δ* mutant (Fig. 4d). On the other hand, an *sgs1-FD* mutation, which disrupts Sgs1 binding to Rad51 and causes a hypo-recombination phenotype (Campos-Doerfler *et al.* 2018), had no effect on the growth of the *sod2Δ* mutant on PQ (Fig. 4d).

Yeast Sod2 activation is dependent on intracellular manganese and iron levels, which are regulated by Smf2 and Mtm1, respectively (Edward *et al.* 2001; Luk *et al.* 2003; Yang *et al.* 2006). To determine if the genetic interaction between *SOD2* and *SGS1* in the suppression of PQ toxicity was indeed dependent on Sod2 function of redox regulation, we performed PQ-hypersensitivity spot assays for the *sgs1Δ* mutant carrying a deletion of *MTM1* instead of *SOD2* and observed increased PQ-sensitivity (Fig. 4e). Although this increase in PQ-sensitivity of the *mtm1Δ sgs1Δ* mutant was not as strong as that of the *sod2Δ sgs1Δ* mutant, probably due to residual Sod2 activity in the *mtm1*Δ mutant, it suggests that the ROS-scavenging function of Sod2 contributes to PQ-tolerance in the *sgs1Δ* mutant.

Using the *CAN1* forward mutation assay (Fig. 4g, Supplementary Table 5), we found that PQ had no effect on the *CAN1* mutation (can^r^) rate of wildtype cells. While the untreated *sod2*Δ mutant had a wildtype can^r^ rate, the rate increased 5-fold upon PQ treatment, suggesting that the pro-oxidant PQ and loss of the mitochondrial antioxidant Sod2 synergize to induce point mutations in the nuclear genome of budding yeast. Introducing TLS mutations, such as deletions of the catalytic subunit of polymerase zeta (*rev3*Δ) or polymerase eta (*rad30*Δ), did not affect the PQ-hypersensitivity of the *sod2Δ* mutant (Fig. 4f), but significantly reduced its high can^r^ rate, with *rev3Δ* and *rev1*Δ mutation having more substantial effects than *rad30*Δ, reducing the PQ-induced can^r^ rate of the *sod2Δ* mutant to wildtype levels (Fig. 4h and Supplementary Table 5). These findings demonstrate that PQ-induced mutagenesis in the *sod2Δ* mutant is primarily controlled by the Rev1/Polζ (Rev3) mutasome.

Despite a strong synergistic interaction between *sod1*Δ and *rad51*Δ in the *CAN1* assay (Choi *et al.* 2018), combining *rad51*Δ with the *sod2*Δ mutation did not cause an increase in the can^r^ rate in the absence of PQ and only a slight increase above additive in the presence of PQ (Fig. 4h). This lack of a genetic interaction between *sod2*Δ and *rad51*Δ in the *CAN1* assay suggests that, unlike in the *sod1*Δ mutant (Choi *et al.* 2018), HR does not suppress small-scale mutations in the *sod2*Δ mutant.

**Fig. 5.**
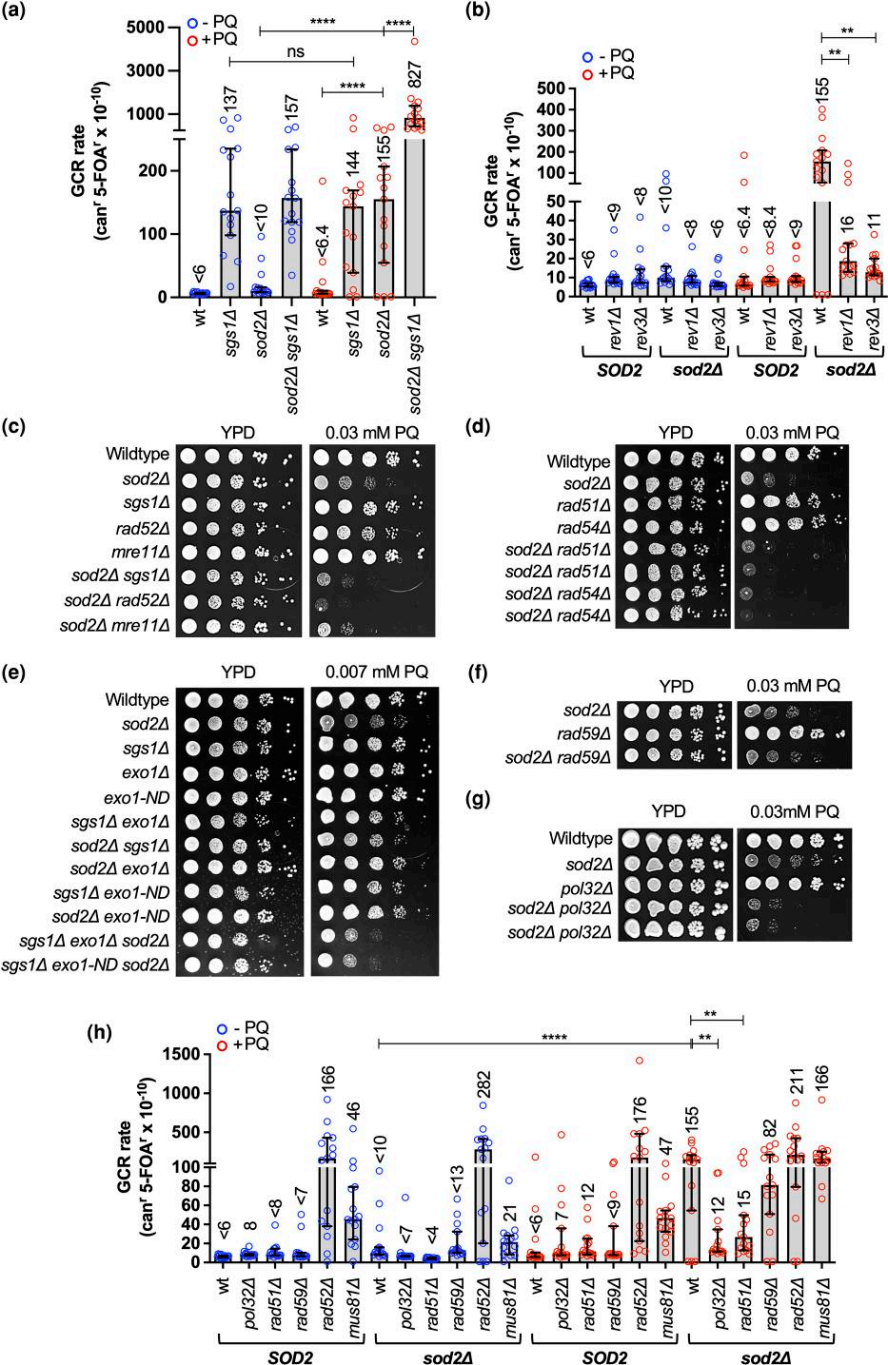
Effect of TLS and the recombination factor Rad51 on PQ-sensitivity and PQ-induced mutator phenotype of the *sod2Δ* mutant. a) Rate of accumulating GCRs in cells harboring *sgs1*Δ and/or *sod2*Δ mutations in the presence or absence of PQ. The GCR rate is the rate of accumulating clones resistant to both canavanine (can^r^) and 5-FOA^r^, which was determined in least 15 cell cultures (open circles) for each yeast strain in the presence and absence of 0.03 mM PQ. Statistical significance of differences was determined by a Mann–Whitney test and reported as ****P ≤ 0.0001; ns, not significant. Numbers above the columns in the graph indicate the median rate of accumulating mutations conferring resistance to canavanine and 5-fluoro-orotic-acid (per cell/generation, can^r^ 5-FOA^r^×10^−10^). For a complete list of all GCR rates with 95% confidence intervals see also Supplementary Table 6. b) Rate of accumulating GCRs in the presence or absence of PQ in the *sod2*Δ mutant harboring a deletion of the TLS genes *REV1 or REV3*. At least 15 cell cultures from 3 isolates for each yeast strain and each condition were analyzed (indicated by open circles). Numbers above the columns in the graph indicate the median rate of accumulating mutations conferring resistance to canavanine and 5-fluoro-orotic-acid (per cell/generation, can^r^ 5-FOA^r^×10^−10^). Statistical significance of differences was determined by a Mann–Whitney test and reported as **P ≤ 0.01. For a complete list of GCR rates with 95% confidence intervals see Supplementary Table 6. c–g) Spot assays to determine PQ sensitivity of the *sod2*Δ mutant with *rad52*Δ, *mre11*Δ, *rad51*Δ, *rad59*Δ, *rad54*Δ, or *pol32*Δ mutations, or *exo1* mutations in the presence or absence of Sgs1. Exo1-ND, nuclease-deficient allele of *EXO1.* Ten-fold-dilutions of exponentially growing cell cultures were spotted on YPD or YPD supplemented with PQ as indicated and incubated at 30°. h) Rate of accumulating GCRs in the presence or absence of PQ in the *sod2*Δ mutant with deletions of *RAD51, RAD52, RAD59*, *MUS81*, or *POL32*. Numbers above the columns in the graph indicate the median rate of accumulating mutations conferring resistance to canavanine and 5-fluoro-orotic-acid (per cell/generation, can^r^ 5-FOA^r^×10^−10^). At least 15 cell cultures for each yeast strain (indicated by dots) and each condition (±PQ) were analyzed. Statistical significance of differences was determined by a Mann–Whitney test and reported as **P ≤ 0.01, ****P ≤ 0.0001. For a complete list of GCR rates with 95% confidence intervals see Supplementary Table 6.

*SGS1* deletion did not further increase the can^r^ rate of the PQ-treated *sod2*Δ mutant (Fig. 4g and Supplementary Table 5), indicating that accumulation of point mutations and other small-scale mutations was not a major contributor to a decreased fitness of the *sod2Δ sgs1Δ* mutant on PQ. Therefore, we measured the rate of GCR formation in the *sod2*Δ and the *sod2Δ sgs1Δ* mutants. The GCR assay measures the rate of functional disruption of 2 counter-selectable marker genes, *CAN1 and URA3,* in the same cell as the result of a chromosome break (Myung *et al.* 2001; Schmidt *et al.* 2006a). We observed a significant (24-fold) increase in the GCR rate of the *sod2*Δ mutant upon treatment with PQ (Fig. 5a and Supplementary Table 6). This rate of PQ-induced GCRs in the *sod2*Δ mutant increased further, by more than 5-fold, upon deletion of *SGS1* (Fig. 5a and Supplementary Table 6), suggesting a novel role for the mitochondrial antioxidant Sod2 in preventing nuclear chromosome rearrangements during exogenous oxidative stress and implicating Sgs1 in suppressing these *sod2*Δ-associated chromosome rearrangements.

Notably, these increases in PQ-induced GCRs in the *sod2*Δ and *sod2Δ sgs1Δ* mutants were not accompanied by increases in Rad52-marked recombinogenic DNA lesions (Supplementary Fig. 5). Considering this lack of Rad52-GFP foci accumulation in the *sod2Δ* mutant and its increased *CAN1* point mutation rate, we reasoned that similar to mismatch-repair-deficient cells (Myung *et al.* 2001) the increased GCR rate in Sod2-deficient cells could be the result of pseudo-GCRs that arise by independent, inactivating point mutations in the *CAN1* and *URA3* genes in the same cell rather than a chromosome rearrangement where a DNA break leads to loss of the nonessential region that contains both *CAN1* and *URA3* (Chen and Kolodner 1999; Schmidt *et al.* 2006a). To distinguish between pseudo-GCRs and classic GCRs, we tested twelve independent GCR clones of the PQ-treated *sod2Δ* mutant for the presence of the *CAN1* and *URA3* genes, revealing that both genes had been lost in 11 clones (Supplementary Fig. 6, a and b), indicative of classic, chromosome-break-associated GCR formation in the PQ-treated *sod2*Δ mutant. Similarly, all fifteen GCR clones obtained from the PQ-treated *sgs1Δ sod2Δ* mutant had lost the *CAN1* and *URA3* genes (Supplementary Fig. 6a), indicating classic GCR formation. These findings support a novel role for the mitochondrial antioxidant Sod2 in the prevention of nuclear chromosome rearrangements during oxidative stress wherein it functionally interacts with Sgs1.

### Chromosomal rearrangements in oxidatively stressed cells depend on TLS and HR

To better understand the mechanism of PQ-induced GCR formation in the *sod2*Δ mutant we tested the effect of disrupting DNA damage repair and tolerance pathways. Disruption of the Rev1/Polζ mutasome (*rev1*Δ, rev3Δ) led to major (10-fold) reductions in the GCR rate of the PQ-treated *sod2Δ* mutant (Fig. 5b, Supplementary Table 6). This was unexpected as the *rev3*Δ mutation previously had no effect on the elevated GCR rate of cells lacking the cytoplasmic antioxidant Tsa1 (Ragu *et al.* 2007), indicating a TLS-dependent mechanism of GCR formation in the PQ-treated *sod2*Δ mutant and a TLS-*in*dependent pathway in the *tsa1*Δ mutant. *POL32*, which codes for the noncatalytic subunit of Polδ that links the polymerase to PCNA, is also a component of Polζ and involved in TLS (Gibbs *et al.* 2005; Pages *et al.* 2008; Baranovskiy *et al.* 2012; Makarova and Burgers 2015). Its deletion also significantly reduced (13-fold) the GCR rate of the PQ-treated *sod2*Δ mutant (Fig. 5h, Supplementary Table 6). However, in contrast to *rev1*Δ and *rev3*Δ mutations, the *pol32*Δ mutation increased the PQ-sensitivity of the *sod2*Δ mutant (Fig. 5g), indicating that Pol32 is also required for increased oxidative stress tolerance of the *sod2*Δ mutant. Besides its role as a subunit of the Polζ mutasome, Pol32 is essential for break-induced replication (BIR) (Lydeard *et al.* 2007), a mutagenic recombination pathway that initiates at one-ended double-strand breaks (DSBs) that arise from collapsed replication forks (Huang *et al.* 2000; Lydeard *et al.* 2007), whereas Pol32 is dispensable for error-free HR pathways and for DNA replication. Together with the observation that *SGS1* deletion increased the PQ-sensitivity of the *sod2*Δ mutant (Fig. 4a) and led to a synergistic increase in the GCR rate of the PQ-treated *sod2*Δ mutant (Fig. 5a), this prompted us to evaluate genetic interactions of the *sod2Δ* mutation with other HR genes.

Mre11 and Rad52 are important HR factors for DSB end resection (Mimitou and Symington 2010) and facilitate Rad51 filament formation (New *et al.* 1998; Song and Sung 2000), respectively. Rad51 and the Rad52 paralog Rad59 are involved in different HR events: Rad51 is involved in synthesis-dependent strand annealing where it performs homology search and strand invasion into a homologous duplex facilitated by Rad54 whereas Rad59 is involved in single strand annealing (SSA), wherein the resected DSB ends anneal at direct repeat sequences, often resulting in interstitial deletions, as well as in some minor pathways of DNA break repair, such as Rad51-independent BIR (Sung 1994; Petukhova *et al.* 1998, 1999; Davis and Symington 2001; Signon *et al.* 2001; Sugawara *et al.* 2003). We observed increased PQ sensitivity of the *sod2Δ* mutant when combined with *rad52*Δ, *mre11Δ*, *rad51*Δ, and *rad54*Δ mutations, but not with *rad59Δ* (Fig. 5, c, d and f), indicating that Rad51-dependent but not Rad59-dependent HR events are required for normal growth of the oxidatively stressed *sod2*Δ mutant. During HR, Sgs1 and Exo1 are both capable of performing long-range resection of DSB ends, but loss of both Sgs1 and Exo1 results in severe resection defects and extremely elevated GCR rates (Gravel *et al.* 2008; Mimitou and Symington 2008; Zhu *et al.* 2008; Doerfler and Schmidt 2014). We found that, unlike an *SGS1* deletion, an *EXO1* deletion had no effect on the growth of the *sod2Δ* mutant on PQ (Fig. 5e), even at higher concentrations (Supplementary Fig. 7a). Only when loss of *SGS1* was combined with loss of *EXO1* or the nuclease-deficient *exo1-ND* mutation did the *sod2Δ* mutant suffer from a severe fitness defect on PQ (Fig. 5e). These findings indicate that Rad51-dependent HR, including steps such as DSB end resection (Sgs1, Exo1), Rad51 filament formation (Rad51, Rad52) and duplex invasion (Rad51, Rad54) contribute to survival of the PQ-treated *sod2*Δ mutant whereas Rad59-dependent events are dispensable.

Since *RAD52* deletion caused GCR accumulation comparable to that of the PQ-treated *sod2*Δ mutant (Fig. 5h, Supplementary Table 6) it was not possible to use Rad52 to further evaluate the role of HR for GCR formation in the PQ-treated *sod2*Δ mutant. However, deletions of *RAD51* or *RAD59* were suitable since they did not cause GCR rate increases in the absence or presence of PQ (Fig. 5h, Supplementary Table 6). *RAD51* deletion significantly lowered the GCR rate of the PQ-treated *sod2Δ* mutant whereas *RAD59* deletion did not (Fig. 5h, Supplementary Table 6), suggesting that Rad51-mediated HR events drive the formation of GCRs in the oxidatively stressed *sod2*Δ mutant. Mus81, a structure-specific endonuclease that cleaves HR intermediates and is important for genome stability (Boddy *et al.* 2001; Hwang *et al.* 2005; Ho *et al.* 2010; Agmon *et al.* 2011; Onaka *et al.* 2020), was dispensable for growth of the *sod2*Δ mutant on PQ (Supplementary Fig. 7b). Notably, despite the established role of Mus81 in suppressing GCRs (Hwang *et al.* 2005; Onaka *et al.* 2020; Muellner and Schmidt 2023), *MUS81* deletion did not increase the GCR rate of PQ-treated cells or the PQ-treated *sod2*Δ mutant (Fig. 5h, Supplementary Table 6). That Mus81 was not required for preventing GCRs in these oxidatively stressed cells suggests that the HR intermediates Mus81 acts on during error-free DNA lesion repair do not arise in these cells. Instead, the GCR analysis suggests that PQ-induced lesions in cells lacking Sod2 are substrates for mutagenic repair mechanisms that require Rad51, Pol32, and the Rev1/Polζ (Rev3) mutasome.

## Discussion

In this study, we have identified a novel function of the mitochondrial antioxidant Sod2 in the suppression of nuclear small-scale mutations as well as GCRs in cells under oxidative stress. Notably, these HR-driven chromosomal rearrangements were dependent on the TLS polymerase Polζ. Sod2 functionally interacts with Sgs1 in the suppression of these GCRs and in tolerating oxidative stress induced by PQ. We also show that cells lacking Sgs1 are hypersensitive to PQ-induced oxidative stress and exhibit elevated ROS levels, increased mitochondrial mass, and increased mitochondrial branching, the latter of which was also inducible by HU-induced replication stress in wildtype cells. Rad52-marked DNA repair centers induced by the absence of Sgs1 were significantly reduced by antioxidant treatment, suggesting that elevated ROS is a source of endogenous DNA lesions in the *sgs1*Δ mutant.

Although Sod2 prevents mitochondrial genome instability (Doudican *et al.* 2005), it was unknown whether this mitochondrial antioxidant contributes to the maintenance of nuclear genome integrity. Unlike Sod1, which relocalizes to the nucleus to act as a transcription factor during oxidative stress (Tsang *et al.* 2014), we show here that Sod2 does not relocalize to the nucleus under oxidative stress, yet suppresses nuclear GCRs under these conditions. The positive (*REV1, REV3, POL32, RAD51*) and negative regulators (*SGS1*) of GCR formation in the PQ-treated *sod2*Δ mutant give us some clues as to the nature of the underlying mechanisms. For example, Sgs1 promotes HR by resecting DSBs and through its physical interaction with Rad51, and it inhibits crossover formation and potentially mutagenic HR events, such as homeologous recombination and BIR (Myung *et al.* 2001; Ira *et al.* 2003; Wu and Hickson 2003; Spell and Jinks-Robertson 2004; Sugawara *et al.* 2004; Lo *et al.* 2006; Gravel *et al.* 2008; Mimitou and Symington 2008; Zhu *et al.* 2008; Jain *et al.* 2009; Lydeard *et al.* 2010; Campos-Doerfler *et al.* 2018). The downregulation of such mutagenic HR events by Sgs1 is likely responsible for the suppression of GCRs by Sgs1 in the PQ-treated *sod2*Δ mutant and would be consistent with Sgs1's known role as a suppressor of GCRs in other contexts (Myung *et al.* 2001; Schmidt *et al.* 2006b). In contrast to Sgs1, Rad51 is required for most HR events, including those that are mutagenic, such as BIR, providing an explanation for the decrease of the GCR rate of the PQ-treated *sod2*Δ mutant upon deletion of *RAD51*.

In addition to HR, we identified TLS polymerases, most notably the Rev1/Polζ mutasome, as a driver of GCR formation in the oxidatively stressed *sod2*Δ mutant. In contrast, GCR formation in cells lacking the antioxidant Tsa1 is not dependent on TLS polymerases (Ragu *et al.* 2007).

Analysis of the critical chromosome V region showed that the GCR clones had not arisen by a high rate of inactivating point mutations in the *CAN1* and *URA3* genes due to PQ treatment of the *sod2*Δ mutant as >90% of the GCRs had lost both ORFs, indicative of chromosome break involvement. While a mechanism by which TLS promotes genome rearrangements is not immediately evident, one possibility is that TLS is needed during DNA synthesis steps of GCR formation, such as those associated with HR, to bypass Polδ-stalling DNA lesions that arise in the PQ-treated *sod2*Δ mutant. Recently, TLS polymerases were shown to drive the formation of complex genome rearrangements when BIR is defective due to the absence of Pif1 (Sakofsky *et al.* 2015). The authors propose a model wherein extension of the invading strand in the BIR bubble stalls, leading to its dissociation and reannealing at a nearby microhomology where DNA synthesis depends on TLS polymerases. An interesting possibility is that the DNA lesions in the PQ-treated *sod2*Δ mutant can be similarly disruptive to DNA synthesis during GCR formation, necessitating an obligatory switch to TLS polymerases before the rearrangement can be resolved into a viable GCR.

The exact nature of the mutagenic damage that contributes to chromosome instability in the PQ-treated *sod2*Δ mutant is not known. PQ induces superoxide radicals and PQ-induced DNA damage includes oxidized bases, especially at pyrimidines, and DNA breaks (Keyer and Imlay 1996; Szabó and Ohshima 1997; Petrovska and Dusinska 1999; Tajai *et al.* 2018), which could be aggravated in the absence of a cellular antioxidant, such as Sod2, leading to increased GCR formation. Through lipid peroxidation, direct or indirect damage to protein structure or activity, or metabolic changes (Fukushima *et al.* 2002; Li *et al.* 2021) PQ could further threaten genome stability indirectly, compounding any direct effects of PQ-induced superoxide radicals on genome integrity.

**Fig. 6.**
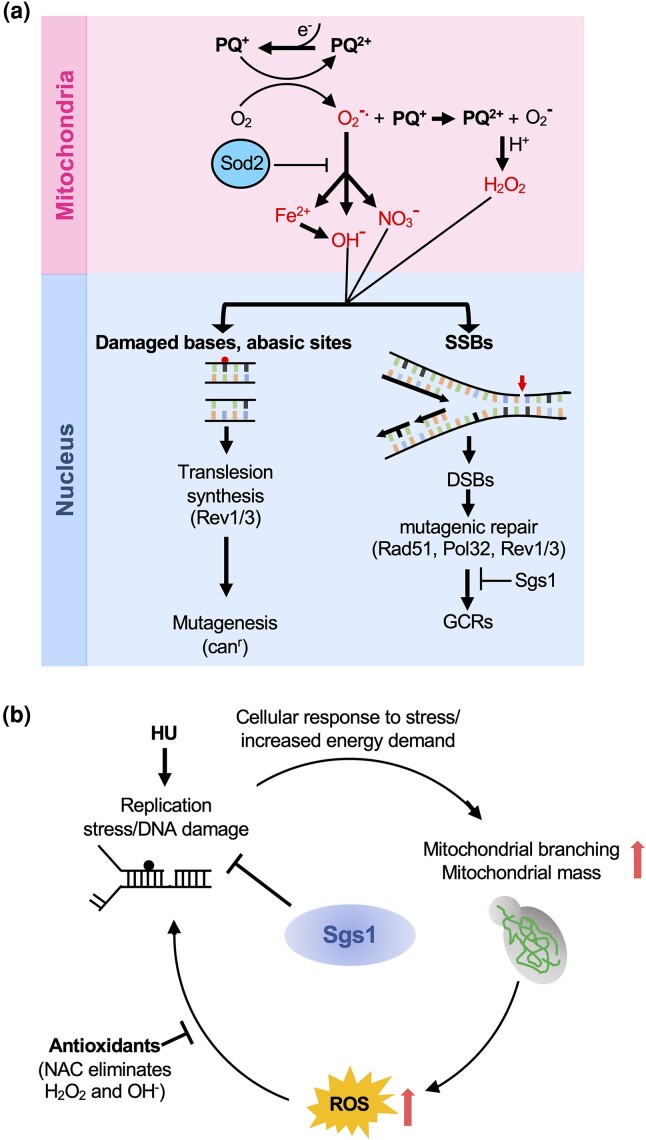
Working models for the role of Sod2 and Sgs1 in the suppression of oxidative stress and genome instability. a) Effects of PQ-induced oxidative stress on genome stability in cells lacking the mitochondrial superoxide dismutase Sod2. The redox cycling drug PQ continuously produces superoxide radicals and additional PQ ions in the mitochondrial matrix, among other cellular locations (Cochemé and Murphy 2008). This uncontrolled superoxide production in the mitochondria can be kept in check by Sod2. However, in the absence of Sod2, superoxide can generate powerful oxidants, such as hydroxyl radicals, peroxynitrite anions, and hydrogen peroxide, and can release free iron from proteins, which can react with other electron donors and produce more ROS like hydroxyl radicals (Keyer and Imlay 1996; Szabó and Ohshima 1997). These ROS can leak out of the mitochondria and result in oxidative DNA damage in the nucleus. Oxidative DNA damage, such as base modifications and abasic sites, the latter of which can be generated spontaneously or as intermediates during BER, can stall the DNA polymerase, requiring TLS to bypass the DNA lesion in an error-prone manner, leading to increased mutagenesis, which can be detected by the *CAN1* forward mutation assay. Hydroxyl radicals and peroxy-nitrite anions directly damage the DNA backbone resulting in DNA SSBs, which can lead to one-ended DSBs. Mutagenic repair of DNA lesions in the PQ-exposed *sod2*Δ mutant by mechanisms dependent on Rad51 and Pol32 can give rise to GCRs. Completion of DNA synthesis steps during GCR formation appears to depend on the bypass of DNA lesions by TLS. In the absence of TLS, replication forks may stall at PQ-induced DNA lesions and abort the mutagenic repair event, thereby suppressing GCRs. These GCR events are suppressed by the antirecombinase Sgs1. b) Hypothesis for the causes and consequences of ROS in cells lacking Sgs1. Loss of Sgs1 results in replication stress and an increased need for DNA repair whose increased demand for ATP induces mitochondrial branching and an increase in mitochondrial mass. This proposed link between the need to respond to the disruption of normal DNA metabolism and the induction of mitochondrial branching is supported by our finding that replication stress induced by HU induces mitochondrial branching similar to the *sgs1*Δ mutant. Increased mitochondrial mass and activity result in an increase in endogenous ROS production and induce additional DNA damage in the *sgs1Δ* mutant, including recombinogenic DNA lesions that can be averted by treatment with the antioxidant NAC.

Based on the new functional interactions of *sod2*Δ in the suppression of nuclear genome instability, we propose the following working model (Fig. 6a): PQ-induced production of superoxide radicals in the mitochondrial matrix can lead to the formation of powerful oxidants (e.g. peroxynitrite, hydroxyl radicals) that can cause oxidative DNA damage in the nucleus that is normally prevented by Sod2, including base damage and DNA single-strand breaks (SSBs). First, increased base damage can stall DNA polymerases, requiring TLS to bypass the lesion, albeit in an error-prone manner, leading to increased mutagenesis. Second, hydroxyl radicals, peroxy-nitrite anions, and hydrogen peroxide can also directly damage the DNA backbone resulting in SSBs. SSBs can be converted into DSBs when a replication fork encounters them and collapses. Mutagenic repair of DNA breaks can result in GCRs whose formation in the *sod2*Δ mutant appears to be homology-directed, as suggested by their promotion by Rad51 and their suppression by Sgs1. Under conditions of elevated oxidative stress in the PQ-exposed *sod2*Δ mutant, the successful completion of DNA synthesis steps of homology-directed repair pathways (Pol32) depends on the successful bypass of oxidative-stress-induced DNA lesions by the Rev1/Polζ mutasome.

In this study, we have also characterized a novel mitochondrial branching phenotype for the *sgs1*Δ mutant based on which we propose a model for the oxidative stress phenotype of cells lacking Sgs1 (Fig. 6b): To help with the response to and repair of DNA lesions induced by the absence of Sgs1, cells induce mitochondrial branching to satisfy their increased energy needs. Mitochondrial branching is associated with increased mitochondrial activity and ATP production (Rudan *et al.* 2018). Notably, replication stress induced by chronic exposure to HU induced the same extensive mitochondrial branching as deletion of *SGS1*, suggesting that increased mitochondrial branching may be a general response of yeast cells with certain DNA metabolic defects or stressors whereby they produce more ATP for increased genome maintenance needs. However, increased mitochondrial activity resulting from increased mitochondrial mass and branching, such as in the *sgs1*Δ mutant, yields increased ROS as a mutagenic byproduct, leading to increased DNA repair need, thus, creating a vicious cycle (Fig. 6b). That mitochondrial branching may be the cause of increased ROS rather than its consequence is supported by our finding that neither the *sod2*Δ mutation nor PQ induced mitochondrial branching. Previous reports of a correlation between recombinogenic DNA lesions mediated by genotoxic stress and an increase in intracellular ROS levels (Yi *et al.* 2016; Choi *et al.* 2018) are also consistent with this model.

While the molecular events that lead to mitochondrial branching in the *sgs1*Δ mutant are unknown, they likely involve a shift from balanced fusion and fission, which generates the tubular mitochondria typical of wildtype cells, toward a state where fusion exceeds fission. Such a shift toward excessive fusion in the *sgs1*Δ mutant could explain why the *sgs1*Δ mutation appears to suppress mitochondrial fragmentation, which is a result of excessive fission, in the *sod2*Δ mutant (Fig. 2b).

In conclusion, we have demonstrated that the mitochondrial antioxidant Sod2 contributes to the maintenance of nuclear genome stability in oxidatively stressed cells by suppressing point mutations and chromosomal rearrangements. That Sod2 did not localize to the nucleus under oxidative stress, unlike Sod1 (Tsang *et al.* 2014), suggests that its contribution to nuclear genome stability may be mediated indirectly through mitochondria-to-nucleus signaling or directly by preventing the accumulation of mitochondrial ROS that could spread to the nucleus, mutate the nucleotide pool, and change the overall steady-state ROS balance in the cell. Since Sod2 is known to suppress mitochondrial genome instability (Doudican *et al.* 2005), mitochondrial DNA abnormalities could also promote nuclear DNA mutagenesis. Indeed, loss of the mitochondrial genome and mitochondrial DNA mutations have been shown to aggravate nuclear genome instability (Rasmussen *et al.* 2003; Veatch *et al.* 2009).

In human cells, pro- and anti-tumorigenic roles of Sod2 have been identified, which appear to be determined by the stage of cancer progression, the type of cancer, and the tumor environment. On one hand, Sod2 is considered a tumor suppressor (Li *et al.* 1995; Zhong *et al.* 1997; Weydert *et al.* 2003; Ough *et al.* 2004; Venkataraman *et al.* 2005; Weydert *et al.* 2006; Thomas and Sharifi 2012), most likely by preventing ROS-induced DNA damage and thereby suppressing tumor initiation, while on the other, pro-metastatic roles of Sod2 have also emerged, for instance in ROS-stress-responsive cancers such as ovarian clear cell carcinoma (Hemachandra *et al.* 2015), where increased Sod2 levels have been found to promote cancer cell migration and invasion and are thought to support the high metabolic activity of cancer cells (Liu *et al.* 2012; Hemachandra *et al.* 2015; Miar *et al.* 2015; Chang *et al.* 2016). Given the oxidative stress phenotype of cells lacking the Bloom's syndrome helicase BLM (Nicotera *et al.* 1989, 1993; Poot *et al.* 1989; Lloret *et al.* 2008; Subramanian *et al.* 2021), the human homolog of yeast Sgs1, increased ROS could contribute to both the initiation and progression of cancers in this highly cancer-prone syndrome (German and Ellis 1998), which would be further enhanced by any changes in Sod2 expression levels.

## Supplementary Material

iyad147_Supplementary_Data

## Data Availability

The authors affirm that all data necessary for confirming the conclusions of the article are present within the article, figures, and tables. Yeast strains and their genotypes are listed in Supplementary Table 1 and are available upon request. The mass spectrometry proteomics data have been deposited to the ProteomeXchange Consortium via the PRIDE (Perez-Riverol *et al.* 2022) partner repository with the dataset identifier PXD040745. Supplementary Table 2 lists all proteins with significant changes identified by mass spectrometry proteomics. Supplemental material available at GENETICS online.
