# Supplementary material for: Mitochondrial superoxide dismutase Sod2 suppresses nuclear genome instability during oxidative stress
Source: Genetics. 2023 Aug 29;225(2):iyad147. doi: 10.1093/genetics/iyad147 (PMC10550321; doi:10.1093/genetics/iyad147)
Supplement: iyad147_Supplementary_Data [file iyad147_supplementary_data.zip › Supplemental_Figures_GENETICS-2023-306108.pdf]

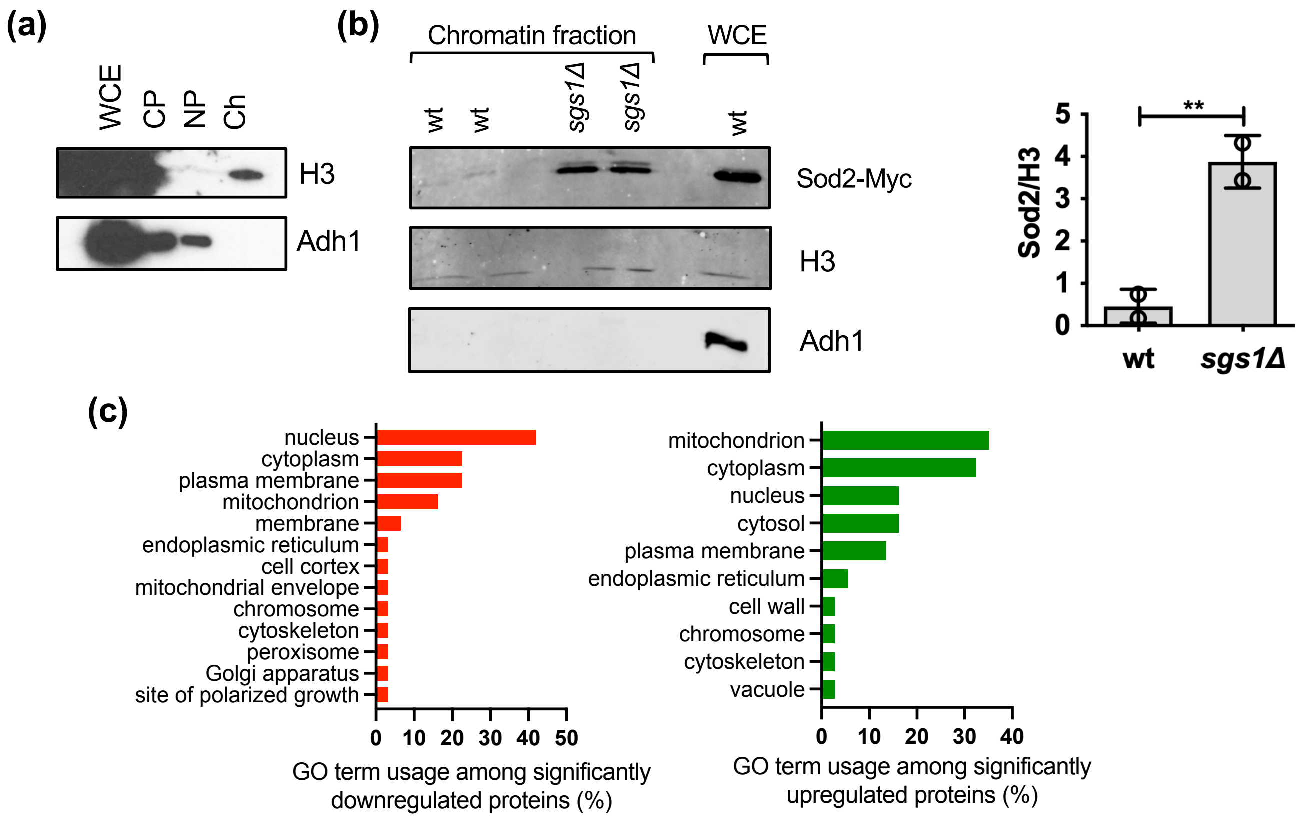

Figure S1. (a) Verification of subcellular fractionation by Western blotting. Whole cell extract (WCE), cytoplasm (CP), nucleoplasm (NP) and chromatin-enriched (Ch) fractions were separated by SDS-PAGE and subjected to Western blotting using Adh1 and histone H3 antibodies. Chromatin enrichment is validated by the presence of histone H3 and absence of cytosolic Adh1. **(b)** Western blot and quantification of Sod2-myc expression levels in two independent chromatin-enriched fractions from wildtype cells (wt) and the *sgs1Δ* mutant. Subcellular fractionation is verified by the presence of histone H3 and absence of Adh1. Whole cell extract (WCE), the first fraction in the chromatin enrichment procedure, from wildtype was added to the Western blot, showing presence of Adh1. Adh1 disappears in the chromatin fraction. There were no differences between Sod2 levels in the WCE of wt and *sgs1Δ* cells (see Fig. 1c). Right panel: The ratio between Sod2.myc and histone H3 is reported with standard deviation. ** $p < 0.01$. **(c)** GO Slim term mapping of the list of proteins from Supplementary Table S2 that were significantly downregulated (left panel) or upregulated (right panel) in the *sgs1Δ* mutant compared to wildtype.

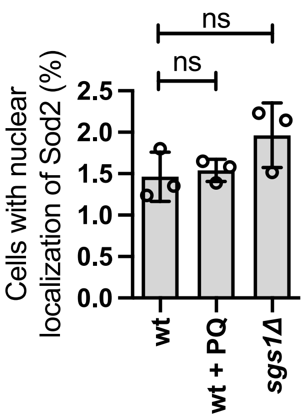

Figure S2. Percentage of cells with apparent overlap between Sod2-RFP and DAPI in unperturbed and PQ-treated wildtype cells, and in unperturbed *sgs1Δ* cells from Fig. 1b. The experiment was performed using three biological replicates (grey dots) and 200-250 cells analyzed by fluorescence microscopy for each replicate. Mean ± SD is reported. Statistical significance was determined with a Student's t-test and reported as: ns, not significant.

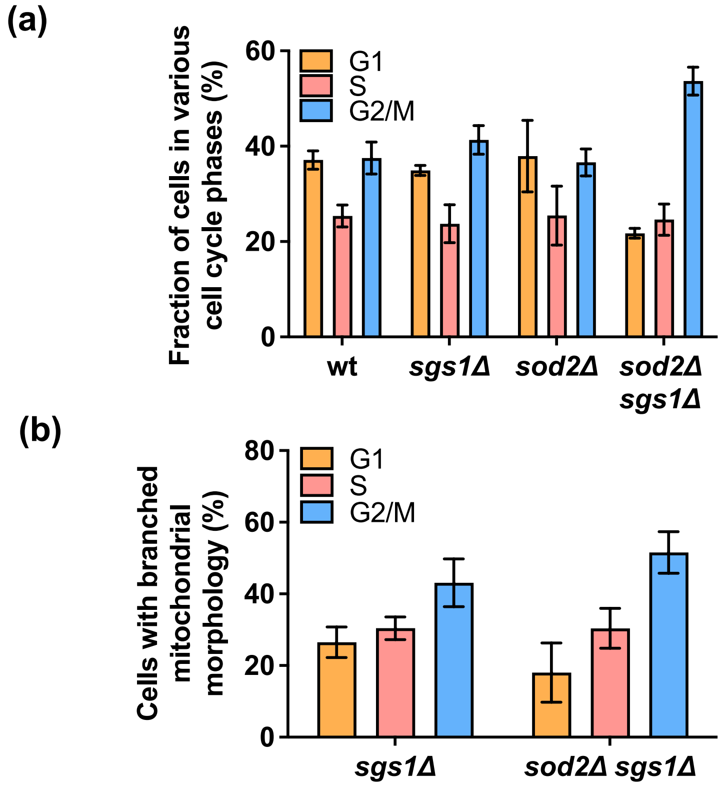

Figure S3. (a) Fraction of cells in G1-phase, S-phase, G2/M phase based on microscopic inspection of bud morphology unperturbed conditions. The experiment was performed using three biological replicates, 100-200 cells were counted for each experiment, and mean % of cells in each cell cycle phase plotted. **(b)** Distribution of cells with branched mitochondrial morphology across the cell cycle phases.

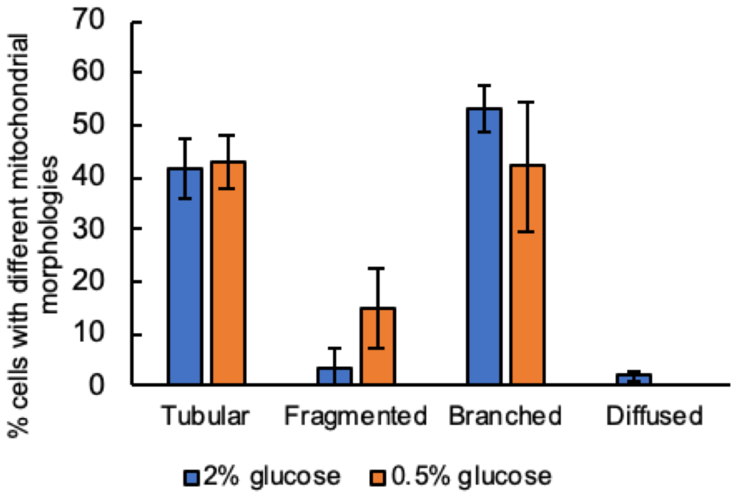

Figure S4. Effect of respiratory activity on mitochondrial branching in the *sgs1Δ* mutant expressing mitochondrial Aco1-GFP. Cells were grown for 18 hours in YPD containing low (0.5%) or high (2%) concentrations of glucose, diluted to OD_{600} of 0.2 in the respective glucose concentrations, grown to OD_{600} of 0.8 and mitochondrial morphology scored by fluorescence microscopy into tubular, fragmented, branched, and diffused. Experiments are performed three times and the mean ± SD is shown.

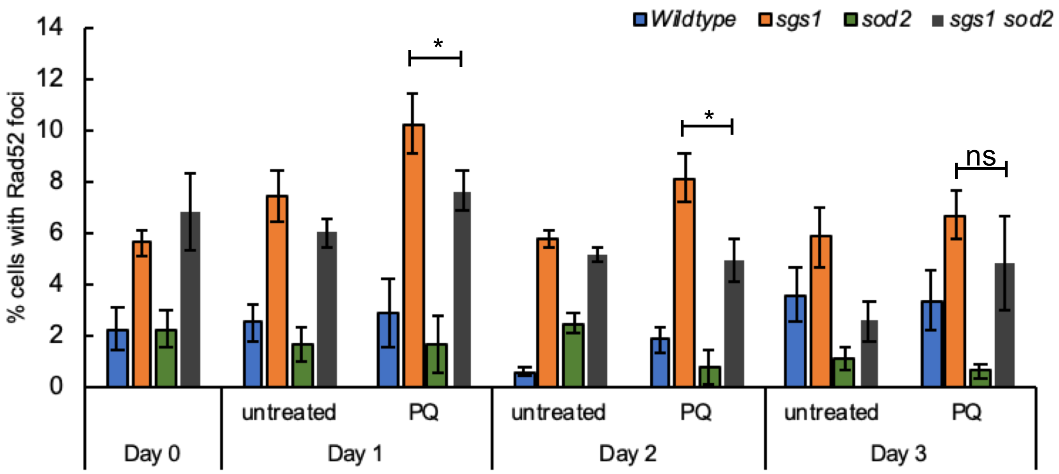

Figure S5. Effect of *sod2Δ* and *sgs1Δ* mutations on accumulation of Rad52 foci in the presence and absence of oxidative stress. Cells expressing GFP-tagged Rad52 were cultured in the absence or presence of 0.03 mM paraquat (PQ), followed by image acquisition on a BZ-X710 fluorescence microscope (Keyence). Cells were cultured for 18 hours in exponential phase (day 0), followed by appropriate dilutions and culturing in the presence or absence of PQ (day 1). This process was repeated twice for a total of three days. Prior to microscopy, cultures were diluted to an OD_{600} of 0.2 and grown to an OD_{600} of 0.8 in the absence or presence of PQ. The experiment was performed with three biological replicates and the mean ± SD is shown. * $p \leq 0.05$; ns, not significant.

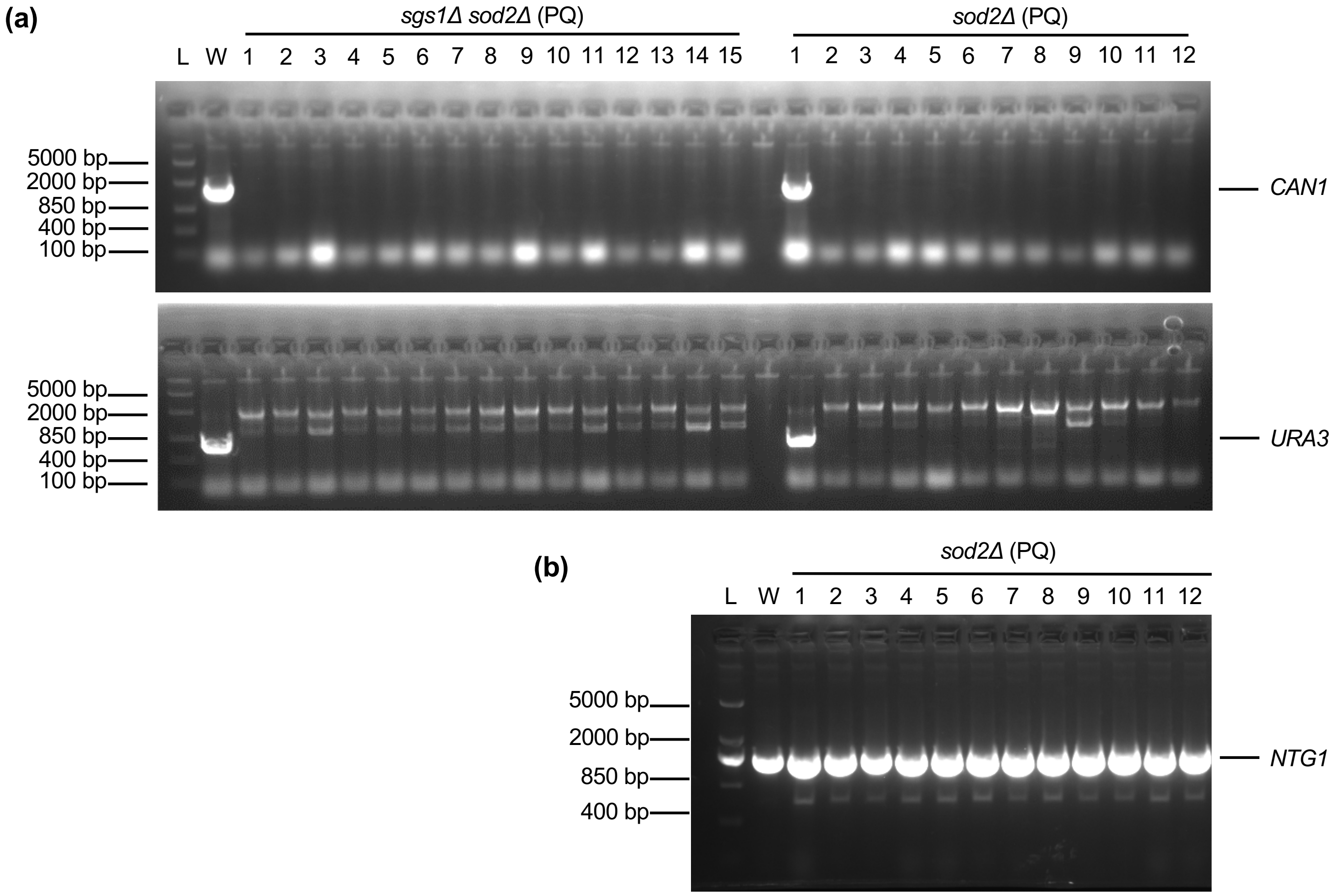

Figure S6. Agarose gel electrophoresis of PCR-amplified *CAN1* and *URA3* genes from GCR clones. ***(a)*** PCR amplification of *CAN1* and *URA3* genes from GCR clones of *sod2Δ* and *sod2Δ sgs1Δ* mutants treated with PQ. Presence of PCR products indicates the presence of the *CAN1* and *URA3* open reading frames, suggesting a point mutation led to gene inactivation. ***b)*** As a technical control for PCR and genomic DNA quality, the open reading frame of the *NTG1* gene was amplified by PCR from genomic DNA used in panel (***A)***. L, DNA ladder, W, wildtype.

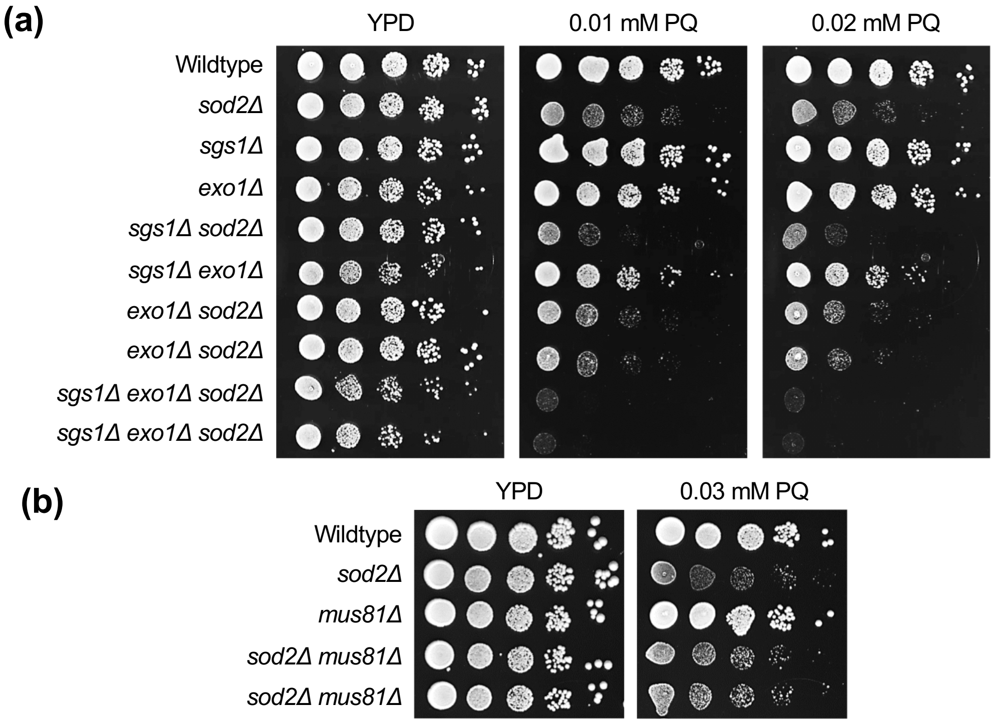

Figure S7. Spot assay to test for genetic interactions of the *sod2Δ* mutation during exposure to oxidative stress. (a) Effect of deletion of *SGS1* and *EXO1* on paraquat (PQ) sensitivity of cells lacking Sod2. **(b)** Effect of deletion of *MUS81* on paraquat (PQ) sensitivity of cells lacking Sod2. Spot assay was performed on exponentially growing cultures by spotting 10-fold dilutions on YPD and on YPD with and without PQ, followed by incubation at 30°C for 2-4 days.
