# Supplementary material for: Mitochondrial superoxide dismutase Sod2 suppresses nuclear genome instability during oxidative stress
Source: Genetics. 2023 Aug 29;225(2):iyad147. doi: 10.1093/genetics/iyad147 (PMC10550321; doi:10.1093/genetics/iyad147)
Supplement: iyad147_Supplementary_Data [file iyad147_supplementary_data.zip › Supplemental_Tables_GENETICS-2023-306108.pdf]

Supplementary Table S1. *Saccharomyces cerevisiae* strains used in this study

Strain ID	Genotype
KHSY802	*MATa, ura3-52, trp1Δ63, his3Δ200, leu2Δ1, lys2bgl, hom3-10, ade2Δ1, ade8, hxt13::URA3*
KHSY1258	*MATa, ura3-52, trp1Δ63, his3Δ200, leu2Δ1, lys2bgl, hom3-10, ade2Δ1, ade8, hxt13::URA3, rad52::HIS3*
KHSY1324	*MATα, ura3-52, trp1Δ63, his3Δ200, leu2Δ1, lys2bgl, hom3-10, ade2Δ1, ade8, hxt13::URA3, sgs1::HIS3*
KHSY1866	*MATa, ura3-52, trp1Δ63, his3Δ200, leu2Δ1, lys2bgl, hom3-10, ade2Δ1, ade8, hxt13::URA3, sgs1-K706A.TRP1*
KHSY2333	*MATa, ura3-52, trp1Δ63, his3Δ200, leu2Δ1, lys2bgl, hom3-10, ade2Δ1, ade8, hxt13::URA3, pol32::loxP-G418-loxP*
KHSY2338	*MATa, ura3-52, trp1Δ63, his3Δ200, leu2Δ1, lys2bgl, hom3-10, ade2Δ1, ade8, hxt13::URA3, exo1::loxP-G418-loxP*
KHSY2752	*MATa, ura3-52, trp1Δ63, his3Δ200, leu2Δ1, lys2bgl, hom3-10, ade2Δ1, ade8, hxt13::URA3, rad51::HIS3*
KHSY2857	*MATa, ura3-52, trp1Δ63, his3Δ200, leu2Δ1, lys2bgl, hom3-10, ade2Δ1, ade8, hxt13::URA3, rad54::HIS3*
KHSY4438	*MATa, ura3-52, trp1Δ63, his3Δ200, leu2Δ1, lys2bgl, hom3-10, ade2Δ1, ade8, hxt13::URA3, sgs1::TRP1, exo1-E150D-D173A.HIS3*
KHSY4460	*MATa, ura3-52, trp1Δ63, his3Δ200, leu2Δ1, lys2bgl, hom3-10, ade2Δ1, ade8, hxt13::URA3, exo1-E150D-D173A.HIS3*
KHSY5109	*MATa, ura3-52, trp1Δ63, his3Δ200, leu2Δ1, lys2bgl, hom3-10, ade2Δ1, ade8, hxt13::URA3, sgs1-F1192D.TRP1*
KHSY5144	*MATa ura3-52, trp1Δ63, his3Δ200, arg4::G418, lys2::HIS3*
KHSY5226	*MATa ura3-52, trp1Δ63, his3Δ200, arg4::G418, lys2::HIS3, sgs1::TRP1*
KHSY5354	*MATa, ura3-52, trp1Δ63, his3Δ200, leu2Δ1, lys2bgl, hom3-10, ade2Δ1, ade8, hxt13::URA3, mre11::HIS3*
KHSY5518	*MATa, ura3-52, trp1Δ63, his3Δ200, leu2Δ1, lys2bgl, hom3-10, ade2Δ1, ade8, hxt13::URA3, Rad52.GFP.HIS3*
KHSY5650	*MATa, ura3-52, trp1Δ63, his3Δ200, leu2Δ1, lys2bgl, hom3-10, ade2Δ1, ade8, hxt13::URA3, mms2::HIS3*
KHSY5730	*MATa, ura3-52, trp1Δ63, his3Δ200, leu2Δ1, lys2bgl, hom3-10, ade2Δ1, ade8, hxt13::URA3, tsa1::TRP1*
KHSY5732	*MATa, ura3-52, trp1Δ63, his3Δ200, leu2Δ1, lys2bgl, hom3-10, ade2Δ1, ade8, hxt13::URA3, sod2::TRP1*
KHSY5752	*MATa, ura3-52, trp1Δ63, his3Δ200, leu2Δ1, lys2bgl, hom3-10, ade2Δ1, ade8, hxt13::URA3, sgs1::HIS3, sod2::TRP1*
KHSY5797	*MATa, ura3-52, trp1Δ63, his3Δ200, leu2Δ1, lys2bgl, hom3-10, ade2Δ1, ade8, hxt13::URA3, Sod2.Myc.TRP1*
KHSY5788	*MATa, ura3-52, trp1Δ63, his3Δ200, leu2Δ1, lys2bgl, hom3-10, ade2Δ1, ade8, hxt13::URA3, sgs1::HIS3, tsa1::TRP1*
KHSY5812	*MATa, ura3-52, trp1Δ63, his3Δ200, leu2Δ1, lys2bgl, hom3-10, ade2Δ1, ade8, hxt13::URA3, sgs1::HIS3, Sod2.Myc.TRP1*

KHSY6114	*MATa, ura3-52, trp1Δ63, his3Δ200, leu2Δ1, lys2bgl, hom3-10, ade2Δ1, ade8, hxt13::URA3, sod2::TRP1, Aco1.GFP.HIS3*
KHSY6128	*MATa, ura3-52, trp1Δ63, his3Δ200, leu2Δ1, lys2bgl, hom3-10, ade2Δ1, ade8, hxt13::URA3, sod2::TRP1, Rad52.GFP.HIS3*
KHSY6138	*MATa, ura3-52, trp1Δ63, his3Δ200, leu2Δ1, lys2bgl, hom3-10, ade2Δ1, ade8, hxt13::URA3, sgs1::HIS3, Rad52.GFP.HIS3*
KHSY6144	*MATa, ura3-52, trp1Δ63, his3Δ200, leu2Δ1, lys2bgl, hom3-10, ade2Δ1, ade8, hxt13::URA3, sod2::TRP1, sgs1::HIS3, Rad52.GFP.HIS3*
KHSY6152	*MATa, ura3-52, trp1Δ63, his3Δ200, leu2Δ1, lys2bgl, hom3-10, ade2Δ1, ade8, hxt13::URA3, sgs1::HIS3, Aco1.GFP.HIS3*
KHSY6160	*MATa, ura3-52, trp1Δ63, his3Δ200, leu2Δ1, lys2bgl, hom3-10, ade2Δ1, ade8, hxt13::URA3, sgs1::HIS3, sod2::TRP1, Aco1.GFP.HIS3*
KHSY6171	*MATa, ura3-52, trp1Δ63, his3Δ200, leu2Δ1, lys2bgl, hom3-10, ade2Δ1, ade8, hxt13::URA3, tsa1::TRP1, sgs1::HIS3, sod2::TRP1*
KHSY6185	*MATα, ura3-52, trp1Δ63, his3Δ200, leu2Δ1, lys2bgl, hom3-10, ade2Δ1, ade8, hxt13::URA3, tsa1::TRP1, sod2::TRP1*
KHSY6196	*MATa, ura3-52, trp1Δ63, his3Δ200, leu2Δ1, lys2bgl, hom3-10, ade2Δ1, ade8, hxt13::URA3, sod2::TRP1, mre11::HIS3*
KHSY6202	*MATa, ura3-52, trp1Δ63, his3Δ200, leu2Δ1, lys2bgl, hom3-10, ade2Δ1, ade8, hxt13::URA3, sod2::TRP1, rad52::HIS3*
KHSY6332	*MATα, ura3-52, trp1Δ63, his3Δ200, leu2Δ1, lys2bgl, hom3-10, ade2Δ1, ade8, hxt13::URA3, sod2::TRP1, sgs1-F1192D.TRP1*
KHSY6335	*MATa, ura3-52, trp1Δ63, his3Δ200, leu2Δ1, lys2bgl, hom3-10, ade2Δ1, ade8, hxt13::URA3, sod2::TRP1, sgs1-K706A.TRP1*
KHSY6355	*MATa, ura3-52, trp1Δ63, his3Δ200, leu2Δ1, lys2bgl, hom3-10, ade2Δ1, ade8, hxt13::URA3, sod2::TRP1, rad51::HIS3*
KHSY6361	*MATa, ura3-52, trp1Δ63, his3Δ200, leu2Δ1, lys2bgl, hom3-10, ade2Δ1, ade8, hxt13::URA3, sod2::TRP1, rad54::HIS3*
KHSY6374	*MATα, ura3-52, trp1Δ63, his3Δ200, leu2Δ1, lys2bgl, hom3-10, ade2Δ1, ade8, hxt13::URA3, sod2::TRP1, exo1::loxP-G418-loxP*
KHSY6379	*MATα, ura3-52, trp1Δ63, his3Δ200, leu2Δ1, lys2bgl, hom3-10, ade2Δ1, ade8, hxt13::URA3, sod2::TRP1, sgs1::HIS3, exo1::loxP-G418-loxP*
KHSY6468	*MATa, ura3-52, trp1Δ63, his3Δ200, leu2Δ1, lys2bgl, hom3-10, ade2Δ1, ade8, hxt13::URA3, rev1::HIS3*
KHSY6635	*MATa, ura3-52, trp1Δ63, his3Δ200, leu2Δ1, lys2bgl, hom3-10, ade2Δ1, ade8, hxt13::URA3, ubc13::TRP1*
KHSY6863	*MATa, ura3-52, trp1Δ63, his3Δ200, leu2Δ1, lys2bgl, hom3-10, ade2Δ1, ade8, hxt13::URA3, Aco1.GFP.HIS3*
KHSY7085	*MATa, ura3-52, trp1Δ63, his3Δ200, leu2Δ1, lys2bgl, hom3-10, ade2Δ1, ade8, hxt13::URA3, rad59::HIS3*
KHSY7086	*MATa, ura3-52, trp1Δ63, his3Δ200, leu2Δ1, lys2bgl, hom3-10, ade2Δ1, ade8, hxt13::URA3, smf2::TRP1*
KHSY7087	*MATa, ura3-52, trp1Δ63, his3Δ200, leu2Δ1, lys2bgl, hom3-10, ade2Δ1, ade8, hxt13::URA3, mtm1::HIS3*
KHSY7088	*MATa, ura3-52, trp1Δ63, his3Δ200, leu2Δ1, lys2bgl, hom3-10, ade2Δ1, ade8, hxt13::URA3, rev3::HIS3*
KHSY7089	*MATa, ura3-52, trp1Δ63, his3Δ200, leu2Δ1, lys2bgl, hom3-10, ade2Δ1, ade8, hxt13::URA3, rad30::HIS3*

KHSY7090	*MATa, ura3-52, trp1Δ63, his3Δ200, leu2Δ1, lys2bgl, hom3-10, ade2Δ1, ade8, hxt13::URA3, mus81::HIS3*
KHSY7091	*MATa, ura3-52, trp1Δ63, his3Δ200, leu2Δ1, lys2bgl, hom3-10, ade2Δ1, ade8, hxt13::URA3, sod2::TRP1, rad59::HIS3*
KHSY7092	*MATα, ura3-52, trp1Δ63, his3Δ200, leu2Δ1, lys2bgl, hom3-10, ade2Δ1, ade8, hxt13::URA3, sod2::TRP1, mus81::HIS3*
KHSY7093	*MATα, ura3-52, trp1Δ63, his3Δ200, leu2Δ1, lys2bgl, hom3-10, ade2Δ1, ade8, hxt13::URA3, sgs1::HIS3, smf2::TRP1*
KHSY7094	*MATa, ura3-52, trp1Δ63, his3Δ200, leu2Δ1, lys2bgl, hom3-10, ade2Δ1, ade8, hxt13::URA3, sgs1::HIS3, mtm1::HIS3*
KHSY7095	*MATa, ura3-52, trp1Δ63, his3Δ200, leu2Δ1, lys2bgl, hom3-10, ade2Δ1, ade8, hxt13::URA3, sod2::TRP1, exo1-E150D-D173A.HIS3*
KHSY7096	*MATa, ura3-52, trp1Δ63, his3Δ200, leu2Δ1, lys2bgl, hom3-10, ade2Δ1, ade8, hxt13::URA3, sod2::TRP1, rev1::HIS3*
KHSY7097	*MATa, ura3-52, trp1Δ63, his3Δ200, leu2Δ1, lys2bgl, hom3-10, ade2Δ1, ade8, hxt13::URA3, sod2::TRP1, rev3::HIS3*
KHSY7098	*MATa, ura3-52, trp1Δ63, his3Δ200, leu2Δ1, lys2bgl, hom3-10, ade2Δ1, ade8, hxt13::URA3, sod2::TRP1, rad30::HIS3*
KHSY7099	*MATa, ura3-52, trp1Δ63, his3Δ200, leu2Δ1, lys2bgl, hom3-10, ade2Δ1, ade8, hxt13::URA3, sod2::TRP1, pol32::loxP-G418-loxP*
KHSY7100	*MATα, ura3-52, trp1Δ63, his3Δ200, leu2Δ1, lys2bgl, hom3-10, ade2Δ1, ade8, hxt13::URA3, sod2::TRP1, sgs1::HIS3, exo1-E150D-D173A.HIS3*
KHSY7101	*MATα, ura3-52, trp1Δ63, his3Δ200, leu2Δ1, lys2bgl, hom3-10, ade2Δ1, ade8, hxt13::URA3, sod2::TRP1, ubc13::TRP1, mms2::HIS3*
KHSY7102	*MATa, ura3-52, trp1Δ63, his3Δ200, leu2Δ1, lys2bgl, hom3-10, ade2Δ1, ade8, hxt13::URA3, sod2::TRP1, ubc13::TRP1*
KHSY7103	*MATα, ura3-52, trp1Δ63, his3Δ200, leu2Δ1, lys2bgl, hom3-10, ade2Δ1, ade8, hxt13::URA3, sod2::TRP1, mms2::HIS3*
KHSY7104	*MATα, ura3-52, trp1Δ63, his3Δ200, leu2Δ1, lys2bgl, hom3-10, ade2Δ1, ade8, hxt13::URA3, ubc13::TRP1, mms2::HIS3*
KHSY7105	*MATa, ura3-52, trp1Δ63, his3Δ200, leu2Δ1, lys2bgl, hom3-10, ade2Δ1, ade8, hxt13::URA3, sgs1::HIS3, Sod2.RFP.HIS3*
KHSY7106	*MATa, ura3-52, trp1Δ63, his3Δ200, leu2Δ1, lys2bgl, hom3-10, ade2Δ1, ade8, hxt13::URA3, Sod2.RFP.HIS3*
KHSY7107	*MATa, ura3-52, trp1Δ63, his3Δ200, leu2Δ1, lys2bgl, hom3-10, ade2Δ1, ade8, hxt13::URA3, sgs1::HIS3, exo1::loxP-G418-loxP*

Supplementary Table S2. Proteins with significant changes in the chromatin-enriched fraction of the *sgs1Δ* mutant

Protein ID	Protein Description	Cellular Component[1]	Fold change[2]
GLK1	Glucokinase-1	C	2.61
RNR2	Ribonucleoside-diphosphate reductase small chain 1	C/N	2.53
PDI1	Protein disulfide-isomerase	ER	2.22
STE23	A-factor-processing enzyme	M	1.94
RNR4	Ribonucleoside-diphosphate reductase small chain 2	C/N	1.89
SOD2	Superoxide dismutase [Mn]	M	1.86
FMP52	Unknown, Found in Mitochondrial Proteome	M	1.72
TCP1	T-complex protein 1 subunit alpha	C	1.70
TSA1	Peroxiredoxin TSA1	C	1.70
CCT6	T-complex protein 1 subunit zeta	C	1.68
SES1	Serine-tRNA ligase	C	1.67
MSS51	translational activator for the mitochondrial COX1 mRNA	M	1.67
BUD3	Bud site selection protein 3	C	1.66
RPS10	40S ribosomal protein S10	C	1.65
NAM7	ATP-dependent helicase NAM7	C	1.65
TPS1	Alpha,alpha-trehalose-phosphate synthase [UDP-forming]	C	1.65
RPS19	40S ribosomal protein S19	C	1.62
YNL208W	Uncharacterized protein	M	1.61
ACO1	Aconitate hydratase	M	1.58
BMH1	14-3-3 protein	C/N	1.58
CDC15	Cell division control protein 15	C	1.56
EDC3	Enhancer of mRNA-decapping protein 3	N	1.56
ALD6	Magnesium-activated aldehyde dehydrogenase	C	1.55
PGK1	Phosphoglycerate kinase	C	1.55
YGL117W	Uncharacterized protein	n.d.	1.55
RPN9	26S proteasome regulatory subunit RPN9	C/N	1.54
ACO2	Homocitrate dehydratase	M	1.54
RPS17	40S ribosomal protein S17	C	1.54
GLN4	Glutamine-tRNA ligase	C	1.52
MMF1	Mitochondrial Matrix Factor	M	1.52
FRS1	Phenylalanine-tRNA ligase beta subunit	C	1.52
PIM1	Lon protease homolog	M	1.52
TIF1	ATP-dependent RNA helicase eIF4A	C	1.52
RPT6	26S protease regulatory subunit 8 homolog	N	1.50
EXG2	Glucan 1,3-beta-glucosidase 2	W	1.50
RPS6	40S ribosomal protein S6	C	1.49
ATP5	ATP synthase subunit 5	M	1.49
GDH1	NADP-specific glutamate dehydrogenase 1	N/C	-0.54
GAT1	Transcriptional regulatory protein	N/C	-0.60
SHM2	Serine hydroxymethyltransferase	C	-0.60
CRP1	Cruciform DNA-recognizing protein 1	N	-0.63

NRD1	RNA-binding subunit of Nrd1 complex	N	-0.64
NHP6B	Non-histone chromosomal protein 6B	N	-0.66
IMD4	Inosine-5-monophosphate dehydrogenase 4	C	-0.67
GLT1	Glutamate synthase [NADH]	M	-0.67
TFC3	Transcription factor tau 138 kDa subunit	M	-0.68
RSC4	Chromatin structure-remodeling complex subunit RSC4	N	-0.68
AST1	Protein AST1	C	-0.69
IMD3	Inosine-5-monophosphate dehydrogenase 3	C	-0.69
TBF1	TTAGGG repeat-Binding Factor	N	-0.70
HXT4	Low-affinity glucose transporter HXT4	C	-0.70
GCV1	Aminomethyltransferase	M	-0.70
QDR2	Quinidine resistance protein 2	C	-0.70
SWI3	SWI/SNF complex subunit SWI3	N	-0.70
SMT3	Ubiquitin-like protein SMT3	N/C	-0.70
RSC8	Chromatin structure-remodeling complex protein RSC8	N	-0.71
MEP2	Ammonium transporter MEP2	C	-0.71
RPB4	DNA-directed RNA polymerase II subunit RPB4	N	-0.71
DPB4	DNA polymerase epsilon subunit D	N	-0.71
NPL6	Chromatin structure-remodeling complex subunit RSC7	N	-0.71
HHO1	Histone H1	N	-0.71
CIT2	Citrate synthase, peroxisomal	C	-0.71
TFB4	RNA polymerase II transcription factor B subunit 4	N	-0.72
YDL183C	Uncharacterized protein	C	-0.72
HSP30	30 kDa heat shock protein	C	-0.72
SWC4	SWR1-complex protein 4	N	-0.72
PUT2	Delta-1-pyrroline-5-carboxylate dehydrogenase	M	-0.72
TFC7	Transcription factor tau 55 kDa subunit	N	-0.73

[1] Based on GO Slim Term Mapping (https://www.yeastgenome.org/goSlimMapper) and Saccharomyces Genome Database, Cellular components: C – cytoplasmic/cytoskeleton/ER/Golgi apparatus/plasma membrane/cell wall; N – nucleus; M – mitochondrion/mitochondrial envelope, n.d. – not determined

[2] The mass spectrometry proteomics data have been deposited to the ProteomeXchange Consortium (http://proteomecentral.proteomexchange.org) via the PRIDE partner repository with the dataset identifier PXD040745.

Supplementary Table S3. Effect of *SOD2* and *SGS1* deletions on the fraction of cells with different mitochondrial morphologies in the presence or absence of paraquat (PQ)

**Genotype**		**Mitochondrial morphology (%)**[1]							
		**Untreated**				**+ PQ**[2]			
		**T**	**B**	**F**	**D**	**T**	**B**	**F**	**D**
wildtype	Mean (%)	81.8	4	10.3	3.7	85.4	5	6.1	2.6
	SD[3]	3.3	2.1	1.2	1.6	1.9	0.9	1.3	1.4
sgs1Δ	Mean (%)	47.9	46.6	3.6	1.9	74.7	7.6	11.7	5.6
	SD	5	1.7	3.4	0.9	6.3	2	4.9	3
sod2Δ	Mean (%)	50.4	1.5	41.5	6.6	51	0.5	33.6	14.9
	SD	5.3	1.4	5.8	3.5	1.4	0.8	4.2	3.5
sgs1Δ sod2Δ	Mean (%)	57.1	32.1	7.9	2.5	41.6	2.6	26.8	29
	SD	4.6	5.8	1.7	0.9	9.9	3.4	3.9	3.2

[1] T – Tubular, B - Branched, F - Fragmented, D - Diffuse
[2] Paraquat (PQ) was added at a concentration of 0.03 mM
[3] SD - Standard Deviation

Supplementary Table S4. Fractions of cells with different mitochondrial morphologies in wildtype cell cultures in the absence or presence of hydroxyurea (HU).

HU (mM)	Major mitochondrial morphology	Mean (%)	SD
0	Tubular	87.5	3.1
	Branched	10	0.9
	Fragmented	0.8	0.8
	Diffuse	1.7	1.7
10	Tubular	79.4	5.3
	Branched	16.8	3.2
	Fragmented	2.2	2.0
	Diffuse	1.5	1.9
200	Tubular	53.9	9.3
	Branched	42.5	9.7
	Fragmented	1.3	0.6
	Diffuse	2.3	1.1

Supplementary Table S5. Effect of mutations in translesion DNA synthesis and homologous recombination on the *CAN1* mutation rate in the *sod2*Δ mutant in the absence and presence of paraquat (PQ)-induced oxidative stress

Relevant genotype	Untreated		+ PQ[1]	
	***CAN1* mutation rate** (can^r x 10^{-8})	**95% CI** (can^r x 10^{-8})	***CAN1* mutation rate** (can^r x 10^{-8})	**95% CI** (can^r x 10^{-8})
wildtype	12	10-17	13	10-15
rev3Δ	3	2-4	4	3-5
rev1Δ	3	2.7-4	3	2.8-5
rad30Δ	8	7-17	10	7-20
rad51Δ	96	83-121	94	88-156
sgs1Δ	15	10-23	16	12-22
sod2Δ	14	11-18	68	57-78
sod2Δ rev3Δ	6	5-7	13	9-16
sod2Δ rev1Δ	6	5-8	24	16-39
sod2Δ rad30Δ	10	7-14	52	38-55
sod2Δ rad51Δ	96	76-135	230	200-300
sod2Δ sgs1Δ	15	11-24	69	45-94

[1] Paraquat (PQ) was added at a concentration of 0.03 mM.

Supplementary Table S6. Effect of *SOD2* deletion and PQ-induced oxidative stress on accumulating gross-chromosomal rearrangements (GCRs)

**Relevant genotype**	**Untreated**		**+ PQ**[1]	
	**GCR rate**[2] (can^r 5-FOA^r x 10^{-10})	**95% CI**[3]	**GCR rate** (can^r 5-FOA^r x 10^{-10})	**95% CI**
wildtype	<6	<5-7	< 6.4	< 5.8-10.5
sgs1Δ	137	98-235	144	39-169
sod2Δ	<10	<8-16	155	55-207
rad52Δ	166	38-430	176	14-482
rad51Δ	<8	<7-15	12	<8-48
rad59Δ	<7.4	<7-10	< 9	<8-38
rev1Δ	<9	<7-10	< 8	<8-10
rev3Δ	<8	<7-14	<9	<8-11
mus81Δ	46	24-80	47	32-55
pol32Δ	8	<7-36	7	<8-36
sgs1Δ sod2Δ	157	119-234	827	439-1380
sod2Δ rad52Δ	282	20-416	211	79-423
sod2Δ rad51Δ	<4	<3-5	15	<13-50
sod2Δ pol32Δ	<6.6	<6-7	12	<11-35
sod2Δ rad59Δ	<10	<5-14	82	51-215
sod2Δ rev1Δ	<8	<7-11	16	<13-28
sod2Δ rev3Δ	<6	<5.5-7	11	<10.5-20
sod2Δ mus81Δ	21	9-28	166	108-253

[1] Paraquat (PQ) was added at a concentration of 0.03 mM.
[2] Gross-chromosomal rearrangement (GCR) rates were calculated from canavanine-resistant (can^r), 5-FOA-resistant (5-FOA^r) colonies (can^r 5-FOA^r x 10^{-10}) as described [1].
[3] 95% confidence intervals (CI) are reported as canavanine-resistant, 5-FOA-resistant colonies (can^r 5-FOA^r x 10^{-10}) and were calculated as described [1,2].

References for supplementary tables

1. Schmidt, K.H., et al., *Analysis of gross-chromosomal rearrangements in Saccharomyces cerevisiae.* Methods Enzymol, 2006. **409**: p. 462-76.
2. Nair, K.R., *Table of confidence intervals for the median in samples from any continuous population.* Sankhya, 1940. **4**: p. 551-558.
